# Ion Exchange Synthesizes a Metastable Layered Polymorph of MgZrN_2_ and MgHfN_2_ Semiconductors

**DOI:** 10.1021/acs.chemmater.4c02748

**Published:** 2025-03-03

**Authors:** Christopher L. Rom, Matthew Jankousky, Maxwell Q. Phan, Shaun O’Donnell, Corlyn E. Regier, James R. Neilson, Vladan Stevanović, Andriy Zakutayev

**Affiliations:** † Materials Science Center, National Renewable Energy Laboratory, Golden, Colorado 80401, United States; ‡ Department of Metallurgical and Materials Engineering, Colorado School of Mines, Golden, Colorado 80401, United States; § Department of Chemistry, Colorado State University, Fort Collins, Colorado 80523-1872, United States

## Abstract

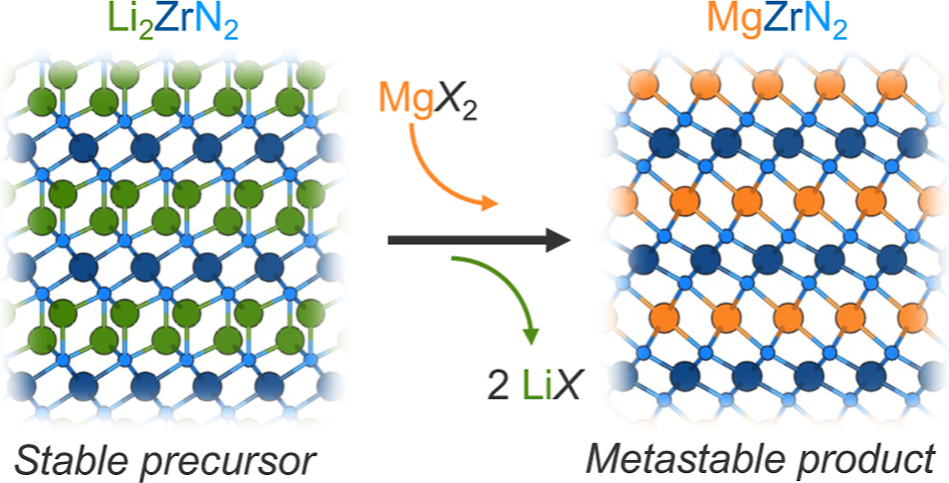

The synthesis of ternary nitride materials is uniquely difficult, in large part because elemental N_2_ is relatively inert. However, lithium reacts readily with other metals and N_2_, making Li-M-N the most numerous subset of ternary nitrides. Here, we use Li_2_ZrN_2_, a ternary nitride compound with a simple synthesis recipe, as a precursor for ion exchange reactions toward AZrN_2_ (A = Mg, Fe, Cu, Zn). In situ synchrotron powder X-ray diffraction studies show that Li^+^ and Mg^2+^ undergo ion exchange topochemically, preserving the layers of octahedral [ZrN_6_]. This reaction yields a metastable layered polymorph of MgZrN_2_ (space group *R*3̅*m*) rather than the calculated ground state structure (*I*4_1_/*amd*). Diffuse reflectance measurements show an optical absorption onset near 2.0 eV, consistent with the calculated bandgap for this polymorph. Our experimental attempts to extend this ion exchange method toward FeZrN_2_, CuZrN_2_, and ZnZrN_2_ resulted in decomposition products (). This experimental outcome is explained by our computational results via the higher metastability of these phases compared to MgZrN_2_. We successfully extended this ion exchange method to other Li-M-N precursors by synthesizing MgHfN_2_ from Li_2_HfN_2_. In addition to the experimental synthesis of metastable *R*3̅*m* polymorphs of MgZrN_2_ and MgHfN_2_, this work highlights the potential of the 63 known Li-M-N phases as precursors to synthesize many other ternary nitride materials.

## Introduction

Developing methods for materials synthesis accelerates the rate at which materials can be discovered. Prof. Francis DiSalvo epitomized this fact, as his development of ammonolysis and Na-flux synthesis methods unlocked many ternary nitrides.^1−5^ Yet ternary nitrides remain underexplored, with reported compounds lagging the number of known ternary oxides by an order of magnitude.^6,7^ Thus, methods must be developed to continue charting this vast phase space.

Recent exploratory syntheses via metathesis reactions or thin film sputtering have discovered many ternary nitrides,^8^ but they often form cation-disordered structures that deviate from the cation-ordered phases predicted by computational studies.^9^ For example, MgZrN_2_ is predicted to form in the γ-LiFeO_2_ structure type (*I*4_1_/*amd*), a cation-ordered variant of the rocksalt structure. However, thin film^10−13^ and bulk metathesis^14,15^ syntheses yielded the cation-disordered rocksalt structure *Fm*3̅*m*. Similarly, ZnZrN_2_ has only been produced via sputtering that yielded cation-disordered rocksalt, *h*-BN, or antibixbyite structures (depending on cation ratios) even though the predicted ground state is a cation-ordered “wurtsalt” structure (with layers of tetrahedral wurtzite-like Zn and octahedral rocksalt-like Zr).^16^ In some cases, rapid thermal annealing can convert thin films with metastable disordered structures to stable ordered structures (e.g., MgMoN_2_, MgWN_2_, ScTaN_2_, MgTa_2_N_3_),^17,18^ but synthesizing metastable ordered structures remains a challenge.

Chemical composition and structure are intimately linked to physical properties. Therefore, improved structural control for ternary nitrides will enable improved property control. As the disordered structures of the ternary nitrides described above differ from the predicted structures,^9^ this results in disagreement between the predicted and observed properties of these materials.^11^ For example, the cation disorder commonly observed in sputtered thin films tends to decrease bandgaps.^19^ In the case of MgZrN_2_, the ordered variant (*I*4_1_/*amd*) is predicted to have an optical bandgap of 2.5 eV, but the disordered *Fm*3̅*m* variant that was experimentally synthesized showed an optical absorption onset of 1.8 eV.^11^ Improving our ability to synthesize cation-ordered polymorphs will therefore improve our ability to precisely realize computationally predicted materials with desired properties.

One strategy for synthesizing cation-ordered ternary nitrides is via ion exchange reactions. In these reactions, precursor cation-ordered ternaries serve as a template whereby the structure of the precursor is largely preserved in the ion-exchanged product. Previously, monovalent ions have been swapped to yield delafossite phases (e.g., NaTaN_2_ + CuI → CuTaN_2_ + NaI)^20−22^ and divalent ions have been exchanged to make nitridosilicates (e.g., Sr_2_Si_5_N_8_ + 2CaCl_2_ → β-Ca_2_Si_5_N_8_ + 2SrCl_2_)^23,24^ More recently, we have shown that two equivalents of a monovalent cation (Li^+^) can be exchanged with one equivalent of a divalent cation (Zn^2+^) to synthesize Zn_3_WN_4_ via Li_6_WN_4_ + 3ZnBr_2_ → Zn_3_WN_4_ + 6LiBr.^25^ That work suggests Li-M-N precursors may hold promise for further heterovalent cation exchange reactions.

Here we report the synthesis of a metastable, cation-ordered polymorph of MgMN_2_ (M = Zr, Hf) in the α-NaFeO_2_ structure type. This metastable polymorph forms via a topochemical ion exchange reaction between Li_2_MN_2_ and MgX_2_ (X = Cl, Br). The layered configuration of the Li_2_ZrN_2_ precursor is retained through the reaction, as observed by in situ synchrotron powder X-ray diffraction. This layered polymorph of MgZrN_2_ exhibits an optical absorption onset of 2.0 eV, consistent with calculations and slightly higher than the rocksalt polymorph (1.8 eV). Our synthetic attempts toward FeZrN_2_, CuZrN_2_, and ZnZrN_2_ instead yielded decomposition products (), which we rationalize via DFT calculations that show these phases are more deeply metastable than MgZrN_2_. This work shows that Li-M-N ternaries are viable precursors for ion exchange syntheses using the examples of MgZrN_2_ and MgHfN_2_, and advances synthesis science by exploring the limits of ion exchange reactions in ternary nitrides.

## Results and Discussion

### Synthesis and Structure of Layered MgZrN_2_

**Figure 1:**
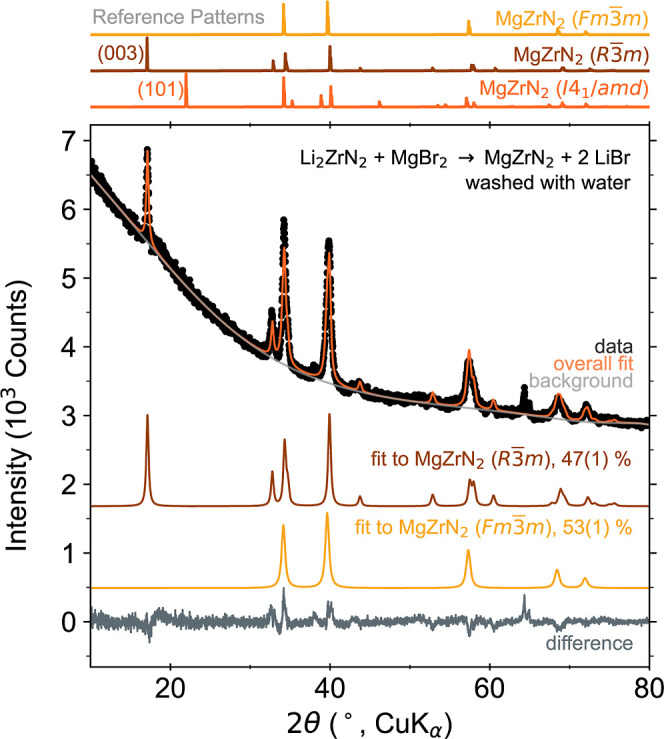
PXRD pattern of MgZrN_2_ synthesized from a reaction between Li_2_ZrN_2_ + MgBr_2_, heated at 650 °C for 10 min. The LiBr byproduct has been washed away with water. Simulated patterns for MgZrN_2_ polymorphs are shown for reference: *I*4_1_/*amd* (mp-1245429), *R*3̅*m* and *Fm*3̅*m* (this work). Peaks of an unknown impurity phase near 64° are not included in the fit.

Reactions between Li_2_ZrN_2_ + MgBr_2_ produce MgZrN_2_ in the α-NaFeO_2_ structure type (space group *R*3̅*m*), along with a LiBr byproduct. Laboratory powder X-ray diffraction (PXRD) measurements indicate that the LiBr could be washed away without changing the MgZrN_2_ structure (Figures 1 and S1). However, synchrotron PXRD collected on the sample after approximately 2 months of air exposure shows evidence of slight moisture sensitivity via the presence of Mg(OH)_2_ (Figure S2). Energy-dispersive X-ray spectroscopy (EDX) measurements on this sample show a slight excess of Mg relative to Zr (Figure S3, Tables S1, and S2), consistent with the presence of both MgZrN_2_ and Mg(OH)_2_. EDX confirms the presence of both N and O, although it cannot reliably determine the ratio of these elements. We therefore refer to this sample as a nitride (MgZrN_2_), but acknowledge that it may be an oxynitride (especially after the water-washing step) and may exhibit off-stoichiometry (Mg_1±*x*_Zr_1±*x*_N_2–*y*_O_*y*_). Using anhydrous solvents may avoid sample degradation during the wash process,^26^ as we had done previously on samples that were more obviously moisture-sensitive.^15,27^ Although EDX cannot detect Li, we identified ca. 1 at % residual Li (metals basis) via quantitative phase analysis on the PXRD data of a sample that was converted to oxides by heating to 900 °C under pure O_2_ (Figure S4, Tables S3, and S4). Future work should optimize reaction conditions to fully remove Li from the nitride phase.

**Figure 2:**
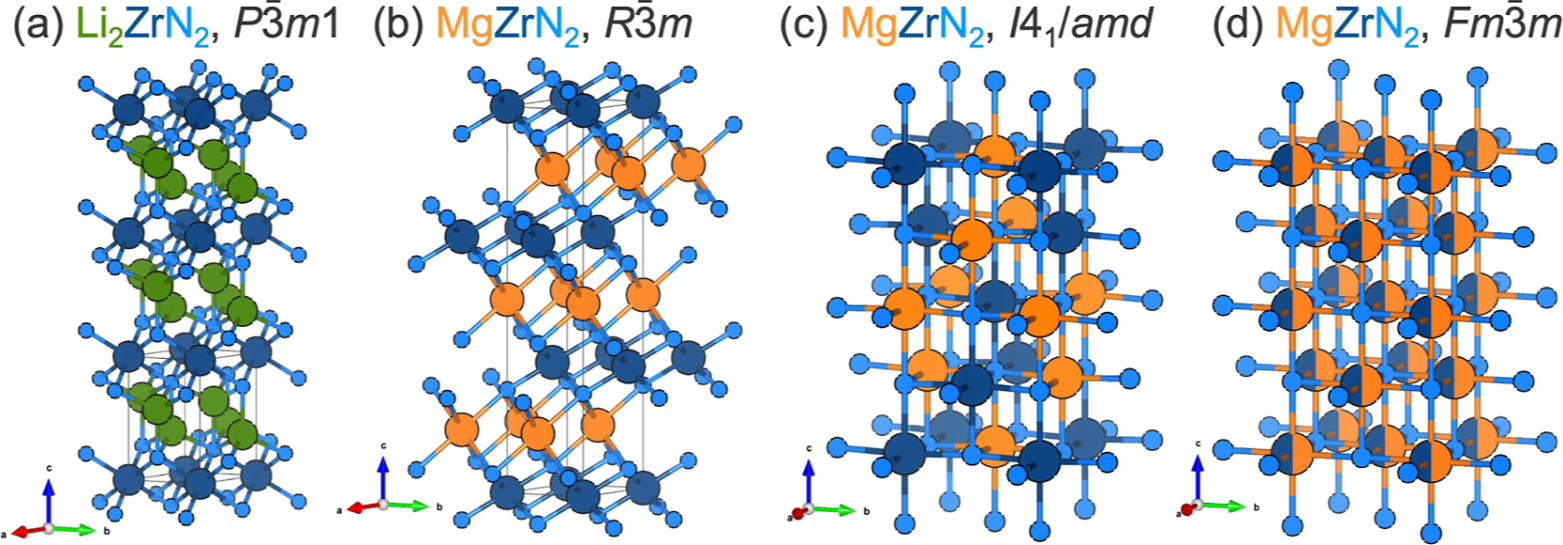
Crystal structures for (a) Li_2_ZrN_2_, *P*3̅*m*1, (b) metastable MgZrN_2_ in the α-NaFeO_2_ structure type, *R*3̅*m*, synthesized here, (c) stable MgZrN_2_ in the γ-LiFeO_2_ structure type, *I*4_1_/*amd*, predicted by theory, and (d) the cation-disordered rocksalt polymorph, *Fm*3̅*m*, reported in prior work.^10−12,14,15^

PXRD prominently shows the (003) reflection of the metastable ordered *R*3̅*m* polymorph of MgZrN_2_, whereas the primary reflection (101) of the stable ordered *I*4_1_/*amd* polymorph does not appear (Figure 1). This indicates that the [ZrN_6_] layers of the Li_2_ZrN_2_ precursor (Figure 2a) are retained in the *R*3̅*m* MgZrN_2_ product (Figure 2b), rather than undergoing a structural rearrangement to the more energetically favorable γ-LiFeO_2_ form (*I*4_1_/*amd*) of cation ordering (Figure 2c). However, the [ZrN_6_] layers shift by *x*+2/3 and *y*+1/3 during the reaction (Figure S5). This shift changes the anion substructure from hexagonal-close-packed (Li_2_ZrN_2_) to cubic-close-packed (MgZrN_2_). The cation substructure is cubic-close-packed for both phases. These changes mean the process is pseudotopotactic rather than strictly topotactic.

Rietveld analysis shows a substantial amount of metal site disorder. In Figure 1, we model this disorder as two separate phases: a fully ordered *R*3̅*m* phase and a fully disordered *Fm*3̅*m* phase, comprising 47(1) mol % and 53(1) mol %, respectively (*R*_wp_ = 4.336%). The lattice parameter of this *Fm*3̅*m* MgZrN_2_ matches prior literature (*a* = 4.54 Å),^10,14^ and is smaller than the parameter for rocksalt ZrN (*a* = 4.58 Å).^28^ Alternatively, the disorder can be modeled as antisite defects using a single *R*3̅*m* phase (*R*_wp_ = 4.709%, Figure S6). This antisite refinement suggests 16% Mg occupancy on the Zr site and vice versa. Similar antisite-defect concentrations were observed in layered LiMoN_2_.^29^ These two approaches to Rietveld produce similar fit qualities, as we also observed for Zn_3_WN_4_ synthesized by a similar ion-exchange process.^25^ This disorder is unsurprising given the identical ionic radii of Mg^2+^ and Zr^4+^ (both 0.72 Å),^30^ and the strong tendency of ternary nitrides toward disordered rocksalt structures.^8,11,16,31,32^ Heating to higher temperatures or for longer times increases disorder, as evidenced by the increase in the fraction of disordered *Fm*3̅*m* MgZrN_2_ relative to the ordered *R*3̅*m* polymorph (Figure S7 and Table S5) and changes the optical properties (Figure S8).

The layered *R*3̅*m* structure of MgZrN_2_ (α-NaFeO_2_-type) that we report here is different from prior experimental and computational reports on this composition. Thin film sputtering experiments^8−13^ and bulk solid state metathesis reactions^14,15^ both produced MgZrN_2_ exclusively in the disordered rocksalt structure *Fm*3̅*m*, along with related solid solutions (Mg_*x*_Zr_2–*x*_N_2_; 0 ≤ *x* ≤ 1). In contrast, two computational studies have identified a *I*4_1_/*amd* polymorph (γ-LiFeO_2_-type) as the thermodynamic ground state,^33,34^ while one group has argued that the γ-LiFeO_2_ ordering (relaxed into a monoclinc LiUN_2_-type structure) and α-NaFeO_2_ ordering are nearly equal in energy.^35^ Both the *I*4_1_/*amd* (γ-LiFeO_2_) and *R*3̅*m* (α-NaFeO_2_) structures are cation-ordered variants of the rocksalt structure (Figure 2).^36^ Our calculations find that the *R*3̅*m* polymorph synthesized here is +42 meV/formula unit higher in energy compared to the ground state *I*4_1_/*amd* polymorph at 0 K. We also calculated the ensemble average structure for the thermodynamic ground state of MgZrN_2_ as a function of temperature (Figure S9). The simulated diffraction patterns for this structure show that the ordered *I*4_1_/*amd* gives way to a disordered *Fm*3̅*m*-like configuration at synthetically relevant temperatures. Increasing heating duration increases the disorder (Figure S7 and Table S5), which supports the computational results that *R*3̅*m* is metastable and kinetically trapped.

Future work to produce fully ordered MgZrN_2_ should account for this tendency toward disorder. Synthesis temperatures and times should be minimized. This may be achieved by choosing reactants to minimize self-heating from the ion exchange,^25^ decreasing reactant particle size to decrease diffusion lengths,^37^ and adding a flux to facilitate reactivity at low temperatures.^4,37^ As MgZrN_2_ is unlikely to be more ordered than the Li_2_ZrN_2_ precursor, maximizing the ordering of this precursor may also be necessary.

### Optical Properties

**Figure 3:**
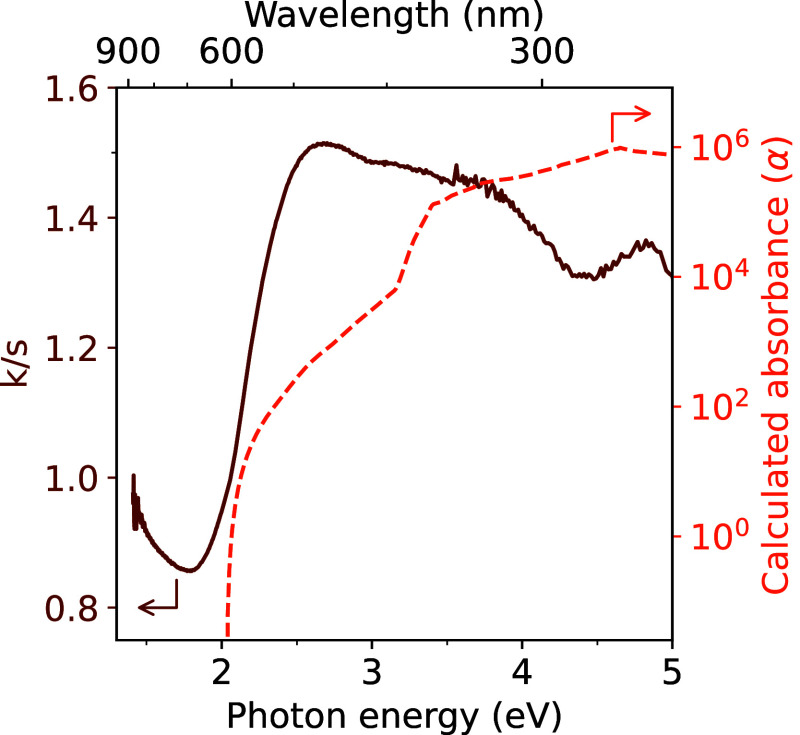
UV–vis diffuse-reflectance spectroscopy measurement for *R*3̅*m* MgZrN_2_ (solid trace) compared with the calculated absorption spectrum (dashed trace).

Optical measurements of the unoptimized *R*3̅*m* MgZrN_2_ synthesized here are consistent with the bandgap predicted in prior literature (Figure 3). The Kubelka–Munk transform of UV–vis diffuse-reflectance spectroscopy on *R*3̅*m* MgZrN_2_ shows an optical absorption onset of 2.0 eV. Similarly, GW calculations show an indirect bandgap of 1.95 eV for the *R*3̅*m* polymorph (NREL MatDB ID #290018),^38,39^ with a direct transition just above 2.0 eV (dashed trace). In contrast, the *I*4_1_/*amd* structure is calculated to have an electronic bandgap of 1.47 eV (NREL MatDB ID #290029) with a direct–but–forbidden transition and an optical bandgap of 2.5 eV.^11^ Thin films of *Fm*3̅*m* MgZrN_2_ (disordered rocksalt) show an absorption onset at 1.8 eV,^10,11^ while *Fm*3̅*m* MgZrN_2_ made via bulk metathesis reactions did not show any clear absorption onset.^14^ Longer annealing times for this synthesis led to a darker powder with a weaker absorption onset (Figure S8). The increased absorbance likely stems from a metallic (or highly defective) phase in the rocksalt structure (e.g., a magnesium-poor Mg_1–*x*_Zr_1+*x*_N_2_, a nitrogen-poor MgZrN_2−δ_, or an oxynitride MgZrN_2−δ_O_δ_), as the structural disorder increased with the darkening of the powder (Table S5). The clear absorption onset for *R*3̅*m* MgZrN_2_ suggests this material may have promising optoelectronic properties for visible light absorption, but further work will be necessary to optimize the synthesis.

### In Situ Synchrotron PXRD

**Figure 4:**
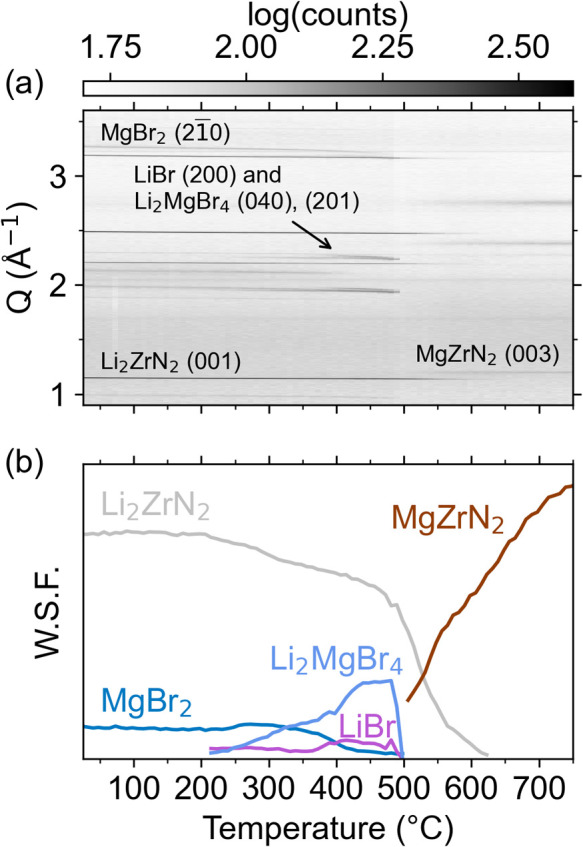
(a) In situ synchrotron PXRD measurements of Li_2_ZrN_2_ + MgBr_2_ as a function of temperature, with select Bragg reflections labeled. (b) Sequential Rietveld analysis of the data shows the relative amounts of crystalline material, expressed as a weighted scale factor (W.S.F.). Select fits are shown in Figure S10.

In situ synchrotron PXRD shows that Li_2_ZrN_2_ undergoes ion exchange with MgBr_2_ at moderate temperatures to produce cation-ordered MgZrN_2_ (Figures 4 and S10). The synchrotron PXRD data were modeled using the Rietveld method to calculate weighted scale factors (W.S.F.) for each phase, which is a proxy for the amount of crystalline material present (see Methods). Initially, Li_2_ZrN_2_ and MgBr_2_ are present, although the MgBr_2_ peaks are fairly weak, possibly owing to poor crystallinity of this precursor. As the temperature increases above 200 °C, reactivity begins, as evidenced by the W.S.F. decreasing for Li_2_ZrN_2_ and the growth of LiBr and Li_2_MgBr_4_ phases. The Li_2_MgBr_4_ is likely an incidental intermediate formed as precursor and product salts react with one another: 2LiBr + MgBr_2_ → Li_2_MgBr_4_. Near 500 °C, the halide peaks disappear, indicating a melting process. At the same time, the MgZrN_2_ peaks grow rapidly and the decline of the Li_2_ZrN_2_ peaks accelerates. The reaction is complete by 640 °C, as the Li_2_ZrN_2_ peaks completely disappear. These diffraction data suggest that the layered structure of Li_2_ZrN_2_ (Figure 2a) templates the layered structure of *R*3̅*m* MgZrN_2_ (Figure 2b).

### Thermochemical Calculations and Comparison with Experiment

The successful synthesis of a metastable, layered polymorph of MgZrN_2_ inspired us to further explore this ion exchange reaction in attempts to synthesize other layered AZrN_2_ compounds (A = Fe, Cu, Zn). DFT calculations show that such compounds are thermodynamically stable against the elements (Table 1). However, in situ synchrotron PXRD (Figures S11–S13) and ex situ PXRD (Figures S14 and S15) experiments show no evidence of FeZrN_2_, CuZrN_2_, or ZnZrN_2_. Instead metallic Fe, Cu, and Zn are observed at low temperatures, indicating nitrogen loss from the solid: Li_2_ZrN_2_ + AX_2_ → A  (where X = Cl, Br). These experiments do not rule out the possibility of synthesizing these phases, but they do suggest that other synthetic methods or conditions may be needed. Only MgZrN_2_ (and MgHfN_2_, see below) exhibited sufficient stability under the temperatures required to drive the ion exchange reactions to completion (ca. 550–650 °C).

**Table 1:** Targeted Compositions and DFT Computed Formation Enthalpies with FERE Corrections to the Chemical Potentials of Elements for AZrN_2_ Phases in the α-NaFeO_2_ Structure Type, along with the Summary of in situ Synchrotron PXRD Reactant Mixtures and Observed Products^a^

target	Δ*H*_f_ (eV/atom)	In situ PXRD data	reactants	observed products
MgZrN_2_	–1.58	Figure 4	MgBr_2_ + Li_2_ZrN_2_	MgZrN_2_, LiBr
FeZrN_2_	–0.91	Figure S11	FeCl_2_ + Li_2_ZrN_2_	Fe, ZrN, LiCl
CuZrN_2_	–0.60	Figure S12	CuBr_2_ + Li_2_ZrN_2_	Cu, ZrN, LiBr
ZnZrN_2_	–0.98	Figure S13	ZnBr_2_ + Li_2_ZrN_2_*	Zn, ZrN, LiBr

a A LiCl/KCl flux was added to the Zn-based reaction to act as a heat sink.

**Figure 5:**
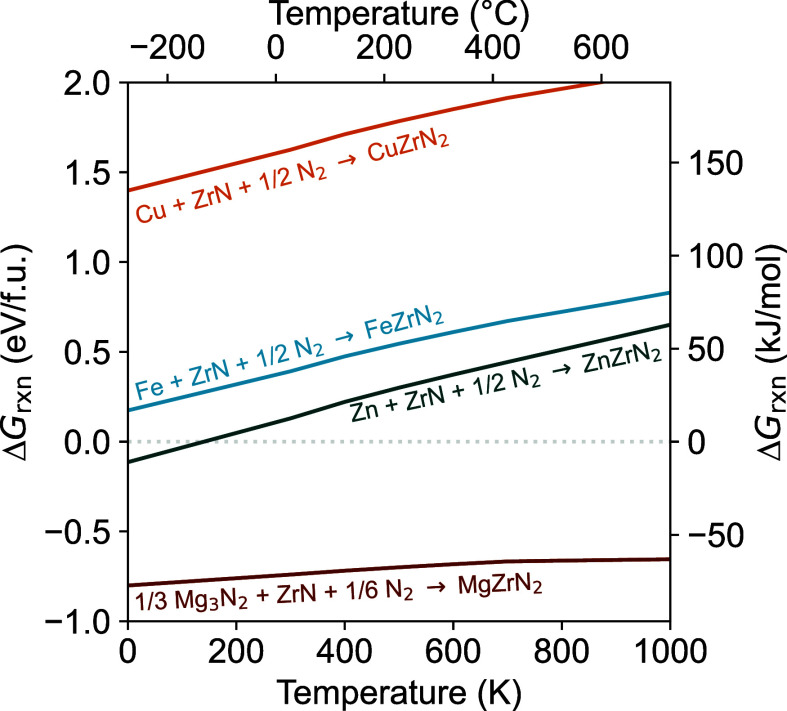
Reaction energies as a function of temperature for AZrN_2_ phases in the α-NaFeO_2_ structure type, compared with elemental or binary competing phases. Positive values indicate thermodynamic instability for the AZrN_2_ phase.

To better understand the thermodynamic (in)stability of these layered nitrides, we calculated Δ*G*_rxn_ as a function of temperature using a machine-learning-derived approximation (Figure 5).^40^ As the overall reactions energies are substantially negative for the Li_2_ZrN_2_ + ZnBr_2_ → ZnZrN_2_ + 2LiBr and Li_2_ZrN_2_ + MgBr_2_ → MgZrN_2_ + 2LiBr reactions (Figure S16), the differing outcomes likely relate to the stability of the AZrN_2_ phases rather than overall Δ*G*_rxn_. Temperature-dependent free energy calculations show MgZrN_2_ is stable against decomposition across the temperature range explored (Figure 5). In contrast, α-NaFeO_2_-type FeZrN_2_, CuZrN_2_, and ZnZrN_2_ are thermodynamically unstable when compared to  at finite temperatures. These calculations are consistent with the in situ diffraction experiments, which show the formation of A + ZrN at low temperatures (Figures S11–S13). We note that a “wurtsalt” structure of ZnZrN_2_ (*P*3*m*1, with layers of tetrahedrally coordinated Zn^2+^) is calculated to be thermodynamically stable,^16^ but we do not observe this phase either (Figure S13).

Although these calculations show that AZrN_2_ (A = Fe, Cu, Zn) phases are not thermodynamically stable at relevant synthesis temperatures, these metastable phases may still be synthesizable in bulk. As the decomposition observed in our reactions targeting AZrN_2_ (A = Fe, Cu, Zn) may stem from the low nitrogen partial pressure in the evacuated capillaries (<30 mTorr), more reactive nitrogen environments may be needed. Woods-Robinson et al. demonstrated that sputtering can synthesize cation-disordered ZnZrN_2_, and that these films were stable up to 300 °C in a vacuum chamber.^16^ Work on another Zn-ternary nitride, Zn_2_NbN_3_, showed that sputtered films did not decompose until >550 °C when annealed under flowing N_2_.^41^ High pressure reactions may also stabilize AZrN_2_ phases, as shown with ZnSnN_2_.^42^ This prior literature suggests that bulk synthesis routes to AZrN_2_ might still be possible, but more work must be done to identify the necessary precursors and reaction conditions to facilitate reactivity at lower temperature or higher pressure.

Layered structures of FeZrN_2_ and ZnZrN_2_ may yet be synthetically accessible. Based on prior literature, ion exchange reactions are capable of synthesizing ternary nitrides that are up to +0.5 eV/f.u. Metastable with respect to decomposition products. For example, metastable delafossite structures of CuMN_2_ (M = Nb, Ta) were synthesized via ion exchange reactions: CuI + NaMN_2_ → CuMN_2_ + NaI.^20,21^ These phases exhibited high metastabilities: Δ*H*_rxn_ = +0.48 eV/f.u. for  → CuNbN_2_, and Δ*H*_rxn_ = +0.42 eV/f.u. for  → CuTaN_2_. The use of iodide anions in these reactions may be essential, as the smaller Δ*H*_f_ for NaI (−287.8 kJ/mol) likely produces less self-heating (and decomposition) compared to the more exothermic reactions yielding NaBr (Δ*H*_f_ = −361.1 kJ/mol) or NaCl (Δ*H*_f_ = −411 kJ/mol).^43^ This trend also holds true for exchanges between Fe/Cu/Zn halides and Li halides (Figure S17). Similar ion exchange reactions in ternary oxides have accessed even more deeply metastable phases such as Sn(Zr_1/2_Ti_1/2_)O_3_ (+0.5 eV/atom; ca. + 2.5 eV/f.u. metastable compared to the binaries).^44^

### Generalizability of Li-M-N Precursors

**Figure 6:**
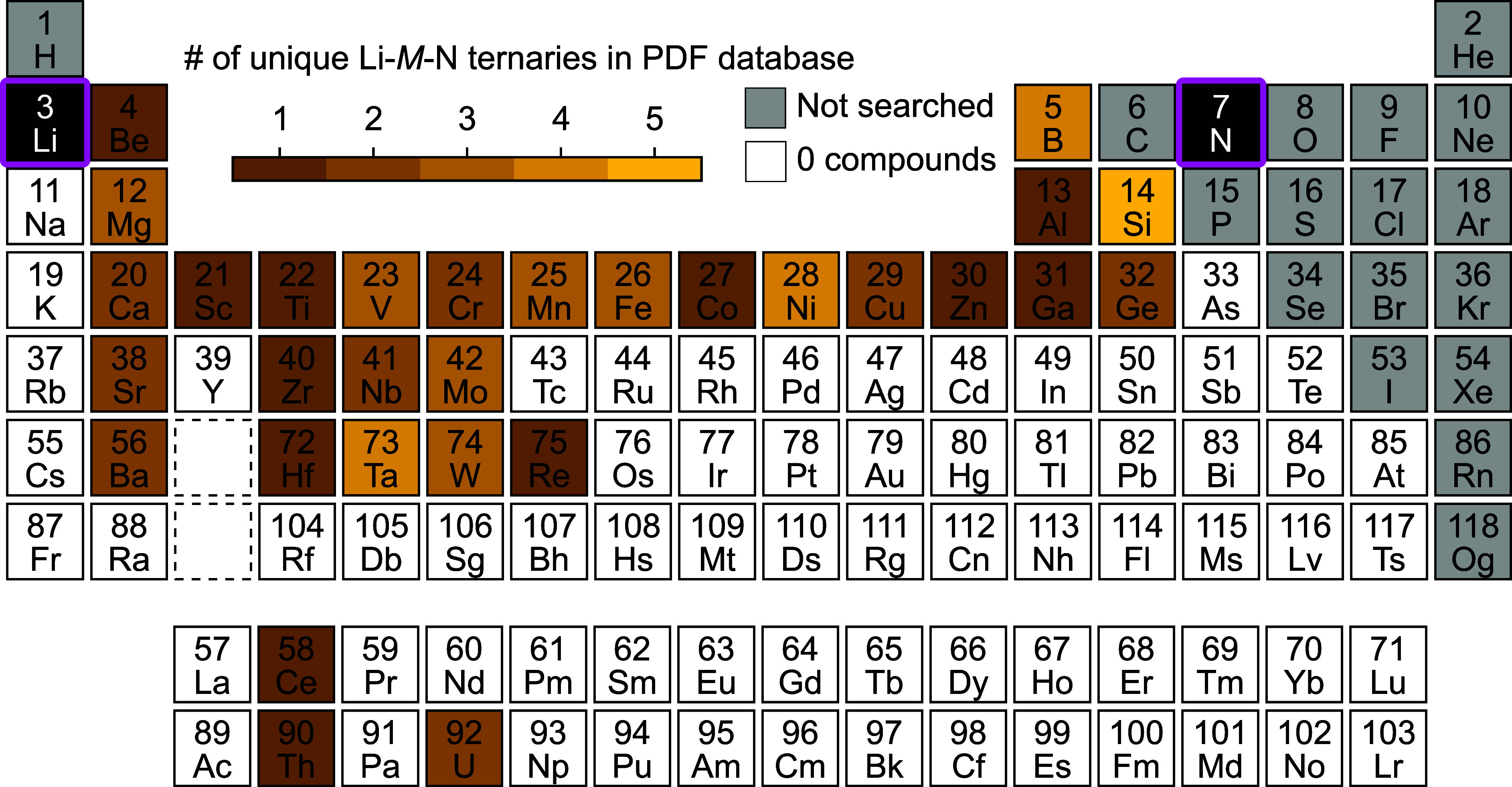
Periodic table of unique Li-M-N ternaries reported in the ICDD PDF database.

**Figure 7:**
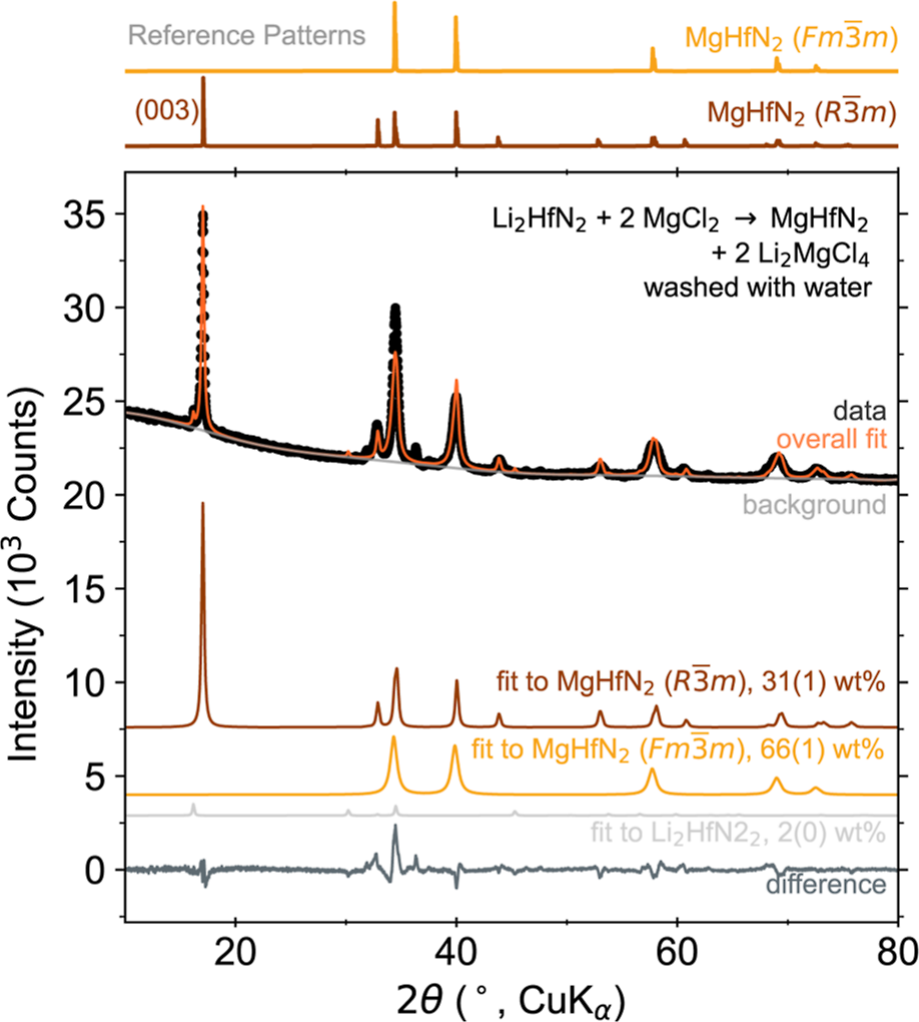
PXRD patterns of MgHfN_2_ synthesized from a reaction between Li_2_HfN_2_ + 2MgCl_2_, heated at 550 °C for 10 h. The Li_2_MgCl_4_ byproduct has been washed away with water. Simulated patterns are shown for reference: MgHfN_2_*Fm*3̅*m* and MgHfN_2_*R*3̅*m*.

Starting with Li-M-N precursors is a generalizable approach for synthesizing ternary nitrides. Of the synthesized ternary nitrides, Li-M-N phases are the most well-studied subclass,^6^ with at least 30 possible options for M (Figure 6, Tables S6 and S7). As a simple example, we synthesized *R*3̅*m* MgHfN_2_ via an ion exchange reaction (Figure 7). We used an excess of MgCl_2_ to drive the reaction Li_2_HfN_2_ + 2MgCl_2_ → MgHfN_2_ + Li_2_MgCl_4_, and subsequently washed the sample with water to remove the halide (Figure 7). While we did not directly compare the effects of using MgCl_2_ vs MgBr_2_, this reaction demonstrates that both precursors are viable. The (003) reflection for the MgHfN_2_*R*3̅*m* structure is prominent. Including preferred orientation in the Rietveld analysis is required to properly fit this peak. We also include a rocksalt MgHfN_2_, which captures some of the site disorder and improves the fit to the peak broadness. The reaction was conducted at 550 °C for a 10 h dwell, which was insufficient to drive full conversion, as indicated by a small amount of residual Li_2_HfN_2_. As Li_2_HfN_2_ is moisture sensitive, we hypothesize that this phase was protected during the washing procedure by a shell of MgHfN_2_. This result demonstrates how these ion exchange reactions may be generalizable for synthesizing other A-M-N phases.

Some elements form multiple Li-*M*-N compounds with different compositions and structures, which may allow for the synthesis of various A-M-N structures. For example, W is known to form Li_6_WN_4_ and LiWN_2_.^45,46^ The Li_6_WN_4_ has tetrahedrally coordinated WN_4_ units in an antifluorite structure,^45^ which we previously used to synthesize the wurtzite-like Zn_3_WN_4_ via a reaction between Li_6_WN_4_ + 3ZnBr_2_.^25^ In contrast, LiWN_2_ is a layered compound with trigonal prismatic WN_6_ units,^46^ and it would be interesting to see if LiWN_2_ can undergo ion exchange to preserve those layers. This example highlights how Li-M-N phases may be useful precursors for rapidly accelerating materials discovery for ternary nitrides.

## Conclusion

We report the synthesis of a metastable polymorph of MgZrN_2_ and MgHfN_2_ in the α-NaFeO_2_ structure type. Powder X-ray diffraction measurements confirmed that cation-ordered nature of this material, which contrasts with prior bulk and thin-film syntheses that produced a cation-disordered rocksalt structure. This synthesized material also differs from prior computational studies that predicted a γ-LiFeO_2_ structure type as the thermodynamic ground state. The synthesized MgZrN_2_ exhibits an optical absorption onset near 2.0 eV, consistent with calculated absorbance spectra for the metastable layered structure *R*3̅*m*. In situ synchrotron powder X-ray diffraction measurements reveal the direct ion exchange pathway from the Li_2_ZrN_2_ + MgBr_2_ precursors to the MgZrN_2_ + 2LiBr products. The key feature of this synthesis is the layered nature of the Li_2_ZrN_2_ precursor, which templates the formation of the layered MgZrN_2_ structure. While first-principles calculations (at *T* = 0 K) suggest that other layered AZrN_2_ phases (A = Fe, Cu, Zn) may be synthesizable, temperature-dependent Δ*G* calculations show that these phases become less stable with increasing temperature. Our in situ X-ray diffraction measurements did not reveal these materials. However, we were able to successfully synthesize layered MgHfN_2_ via this ion exchange method, demonstrating how Li-M-N phases may be used as precursors to make other A-M-N compounds. These results show that ion exchange reactions are a promising strategy for improved structural control in the synthesis of emerging ternary nitride semiconductors.

## Methods

### Synthesis

#### Synthesis of Li_2_MN_2_ Precursors

The synthesis of Li_2_MN_2_ (M = Zr, Hf) was adapted from prior reports.^47,58^ For Li_2_ZrN_2_, Li_3_N (Sigma-Aldrich, ≥99.5%, 60 mesh) and ZrN (Alfa Aesar, 99%) were combined (ca. 1.3:1 mol ratio, ca. 100% excess Li_3_N), homogenized with an agate mortar and pestle, loaded into a Zr crucible with a Zr lid, placed in an open quartz ampule, loaded in a quartz process tube, and transferred from the glovebox to the furnace without air exposure using custom self-sealing end-caps (Figure S18a). The samples were heated under flowing nitrogen (60 sccm, 99.999% purity) at +10 °C/min to 900 or 1000 °C, dwelled for between 3 and 12 h, cooled naturally, and recovered into the glovebox. Excess Li_3_N volatilized away from the sample and reacted with the sacrificial quartz ampule. Regrinding (sometimes with small additions of Li_3_N) and reheating were necessary to achieve phase purity, as ZrN and an unidentified (but presumably Li-rich) phase were often observed owing to variable Li_3_N evaporation rates. For Li_2_HfN_2_, a similar method was used, but Hf powder (Goodfellow, 95%) was used as a precursor rather than the binary nitride.

#### Synthesis of MgMN_2_

Samples were prepared by mixing Li_2_MN_2_ + MgX_2_ (X = Cl, Br), homogenizing in an agate mortar and pestle, compressing ca. 100 mg pellets (0.25 in. diameter) at approximately 1 ton pressure, and heating. Anhydrous halides were used: MgCl_2_ (Sigma-Aldrich, 99.99%, AnhydroBeads) and MgBr_2_ (Thermofisher, 98%, anhydrous). Samples were sealed in quartz ampules under vacuum (<30 mTorr) and heated in muffle furnaces at temperatures and times specified in the text (Figure S18b). Ramp rates were either +5 °C/min or +10 °C/min, and samples were allowed to cool naturally in the furnace. Samples were recovered into an argon glovebox for initial PXRD. To remove the halide byproduct, samples were removed from the glovebox and washed three times with water and once with isopropanol. The resulting powders were generally brick red to dark gray, depending on synthesis conditions.

### Characterization

#### Ex Situ PXRD

The products of all reactions were characterized by powder X-ray diffraction (PXRD). Laboratory X-ray diffraction patterns were collected on a Rigaku Ultima IV diffractometer with Cu Kα X-ray radiation at room temperature. All samples were initially prepared for PXRD measurements inside the glovebox; powder was placed on off-axis cut silicon single crystal wafers to reduce background scattering and then covered with polyimide tape to impede exposure to atmosphere. After laboratory PXRD suggested that MgZrN_2_ was moderately air stable, PXRD patterns were collected without polyimide tape to decrease the background signal and samples were stored in air.

Ex situ synchrotron PXRD measurements were conducted at beamline 2–1 of the Stanford Synchrotron Radiation Lightsource (SSRL) with a 17 keV X-ray energy (λ = 0.7293 Å). Washed samples of MgZrN_2_ were sieved (no. 100, 0.15 mm pore size) to ensure a fine powder, loaded into 0.5 mm OD (0.01 mm wall thickness) quartz capillaries, tamped down with a thin steel wire, and flame-sealed under vacuum.

#### Compositional Analysis

Energy-dispersive X-ray spectroscopy (EDX) was performed using a ThermoFisher Pathfinder EDX in a Hitachi S4800 Scanning Electron Microscope (20 kV accelerating voltage, 10 μA current). Atomic % values were averaged from 3 large-area mapping spectra conducted on a pelletized sample of washed MgZrN_2_.

#### In Situ Synchrotron PXRD

In situ synchrotron PXRD experiments were conducted at beamline 2–1 of the Stanford Synchrotron Radation Lightsource (SSRL) with a 17 keV X-ray energy (λ = 0.7293 Å). Reactant mixtures were loaded into 0.5 mm OD (0.01 mm wall thickness) quartz capillaries and flame-sealed under vacuum. These capillaries were subsequently nested inside 1.0 mm OD quartz capillaries (0.01 mm wall thickness) and mounted in an Anton Paar HTK 1200N heating stage. The capillary was rotated with a frequency of 1 Hz during the experiments. Diffraction patterns were collected with a small area detector (Pilatus 100 K) at a 700 mm sample-to-detector distance. Each 1D pattern was stitched together from 15 separate 2D exposures (1 s each) at 2° steps between 6 and 36° 2θ, with radial integration and merging conducted on-the-fly with custom python code. One full pattern was collected every 50 s, accounting for motor movements.

#### Analysis of Diffraction Data

Ex situ PXRD patterns were analyzed using TOPAS Professional v6.^48^ To create the MgZrN_2_ and MgHfN_2_ structures in space group *R*3̅*m*, α-NaFeO_2_ was used as a starting point (ICSD Collection Code 187705). Na was replaced with Mg, Fe was replaced with Zr (or Hf), and O was replaced with N. Subsequently, this model was refined against the data by first fitting lattice parameters, then crystallite size broadening (Lorentzian), then displacement parameters (*B*_iso_). The background was modeled with a 10-term polynomial. Peak intensities and peak shapes were poorly captured by the fully ordered *R*3̅*m* phase alone. An improved fit was obtained by a two-phase model: the *R*3̅*m* phase was fixed as fully cation-ordered and a fully disordered rocksalt MgMN_2_ phase was added to the model.^14^ Alternatively, a single-phase refinement was performed for comparison using the *R*3̅*m* structure: we refined Mg site occupancy such that Mg refined as 1 – *x* and Zr refined as *x*, and vice versa for the Zr site. In the case of MgHfN_2_, the (003) reflection of the *R*3̅*m* phase was under-fit unless a preferred orientation term was used.

Sequential Rietveld refinements were conducted on in situ SXPRD data sets using TOPAS Professional v6.^48^ Lattice parameters, background terms, and scale factors were refined for each phase as a function of temperature, while atomic coordinates and occupancies were held constant at the initial values of the reference structure. A weighted scale factor (W.S.F.) *Q* was calculated for each phase *p* as a product of scale factor *S*, cell volume *V*, and cell mass M: *Q*_p_ = *S*_p_·*V*_p_·*W*_p_. We note that amorphous and liquid phases are inherently not observed in powder diffraction measurements and therefore cannot be accurately included in this analysis. A Lorentzian size broadening term was refined for each phase to model the peak shape using the pattern showing the greatest intensity of the relevant phase; this term was then fixed for the sequential refinements to better account for changes in intensity. To help stabilize the sequential refinement, isotropic displacement parameters (*B*_iso_) were fixed at 1 Å^2^ for all atoms, but we note that this is likely not physical for a variable temperature investigation.

#### Optical Measurements

UV–vis measurements were conducted on a Cary 6000 UV–vis–NIR spectrometer. BaSO_4_ was used as a white reflectance standard. Absorbance was calculated with the Kubelka–Munk transformation, *k*/*s* = (1 – *R*)^2^/2*R* where *R* is the reflectance, *k* is the apparent absorption coefficient, and *s* is the apparent scattering coefficient.

### Computational Methods

The total energies of ternary nitrides and their decomposition products were calculated using density functional theory (DFT)^49^ as implemented in the Vienna ab initio Simulation Package (VASP).^50^ The formation enthalpy for each compound was computed using fitted elemental-phase reference energies (FERE) to correct the chemical potentials.^51^ These calculations were performed using the generalized gradient approximation (GGA) with the Perdew–Burke–Ernzerhof (PBE) exchange–correlation functional.^52^ Atomic cores were modeled using projector-augmented-wave (PAW) pseudopotentials.^53^ The pseudopotentials were those used in the FERE standard. To converge the total energy with the relatively hard nitrogen pseudopotential used in this standard, a plane-wave cutoff energy of 450 eV was used. The k-point grids were generated automatically with 20 subdivisions along each reciprocal lattice vector. Spin degrees of freedom were treated explicitly for Fe and Cu atoms, testing a ferromagnetic configuration as well as several antiferromagnetic configurations in the α-NaFeO_2_ structure, and using the lowest energy spin configurations when calculating reaction energies. For each composition, structure, and spin configuration, the atomic positions and the shape and volume of the primitive cell were optimized using the conjugate gradient algorithm. Repeated relaxations of cell shape and volume were performed for numerical reasons. Each relaxation is considered converged when the difference in total energy between steps is less than 10^–6^ eV. Gibbs free energies of formation as a function of temperature are calculated using a descriptor for the vibrational entropy contribution developed by Bartel et al.^40^ Symmetry inequivalent cation occupations and their degeneracies for unit cells with 16 atoms were identified using AFLOW.^54,55^ The probabilities of each configuration at different temperatures were then calculated as *p*_i_ = (1/*Z*)*g*_i_* exp(−Δ*E*_*i*_/*kT*), where *Z* = ∑_i_*p*_i_ is the partition function, *g*_*i*_ is the degeneracy of the configuration, and Δ*E*_i_ is the energy of the configuration above the ground state γ-LiFeO_2_. Diffraction patterns were generated for each configuration, and are ensemble averaged with the weights *p*_*i*_.

## Supporting Information Available

Compositional analysis by EDX, ex situ PXRD experiments on MgZrN_2_, UV–vis experiments on MgZrN_2_, in situ synchrotron PXRD experiments toward AZrN_2_ phases, ex situ synthetic attempts toward ZnZrN_2_, ion exchange thermodynamics and Li-M-N entries in the PDF 2021 database, and additional references^56,57^ (PDF)
Cation ordered *R*3̅*m* structure of MgZrN_2_ (CIF)
Cation ordered *R*3̅*m* structure of MgHfN_2_ (CIF)
Cation disordered *Fm*3̅*m* structure of MgZrN_2_ (CIF)
Cation disordered *Fm*3̅*m* structure of MgHfN_2_(CIF)

## Supplementary Material

cm4c02748_si_001.pdf

cm4c02748_si_002.cif

cm4c02748_si_003.cif

cm4c02748_si_004.cif

cm4c02748_si_005.cif
