# Supplementary material for: Ion Exchange Synthesizes a Metastable Layered Polymorph of MgZrN2 and MgHfN2 Semiconductors
Source: Chem Mater. 2025 Mar 3;37(6):2136–44. doi: 10.1021/acs.chemmater.4c02748 (PMC11948327; doi:10.1021/acs.chemmater.4c02748)
Supplement: Supplementary file 1 — cm4c02748_si_001.pdf [file cm4c02748_si_001.pdf]

Supporting Information for: Ion exchange synthesizes a metastable layered polymorph of $MgZrN_2$ and $MgHfN_2$ semiconductors

Christopher L. Rom,[*,†] Matthew Jankousky,[‡] Maxwell Q. Phan,[†] Shaun O'Donnell,[†] Corlyn E. Regier,[¶] James R. Neilson,[¶] Vladan Stevanović,[‡,†] and Andriy Zakutayev[*,†]

†*Materials Science Center, National Renewable Energy Laboratory, Golden, CO, 80401, USA*

‡*Department of Metallurgical and Materials Engineering, Colorado School of Mines, Golden, Colorado 80401, USA*

¶*Department of Chemistry, Colorado State University, Fort Collins, CO, 80523-1872, USA*

E-mail: christopher.rom@nrel.gov; Andriy.Zakutayev@nrel.gov

Contents

Additional XRD experiments, structural analysis, and compositional analysis on $MgZrN_2$ S2

Additional UV-vis experiments on $MgZrN_2$ S10

Ensemble calculations for $MgZrN_2$ S11

***In situ* synchrotron PXRD experiments S12**

***Ex situ* synthetic attempts towards $ZnZrN_2$ S17**

Ion exchange thermodynamics S19

Li-M-N entries in the PDF 2021 database S22

Synthesis set-up S31

Additional XRD experiments, structural analysis, and compositional analysis on $MgZrN_2$

The $MgZrN_2$ samples did not exhibit obvious signs of degradation after exposure to air and water. Figure S1 shows patterns for the as-synthesized $Li_2ZrN_2 + MgBr_2 \longrightarrow MgZrN_2 + LiBr$ (protected from air and moisture with polyimide tape) compared to the XRD pattern of the same sample after washing with water. In the as-synthesized sample, the large background scattering of the polyimide tape obscures the (003) reflection of the $MgZrN_2$ phase. Washing clearly removes the LiBr phase, and does not shift the peaks of the $MgZrN_2$ phase. As the washed sample was measured in air (without polyimide tape), the (003) reflection appears prominently.

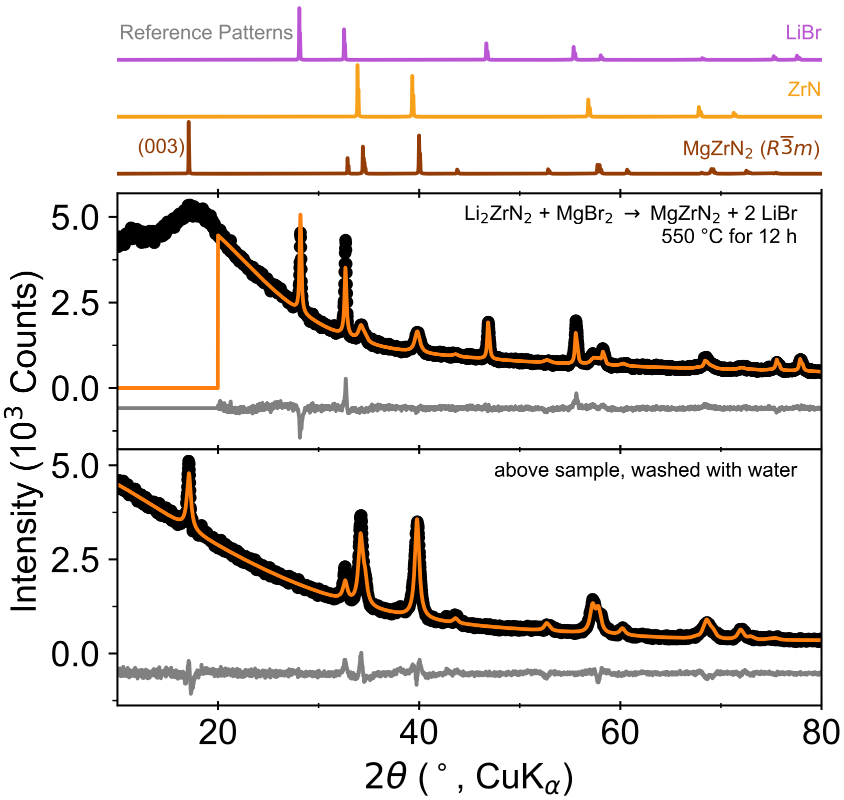

Figure S1: PXRD of the reaction product between $Li_2ZrN_2 + MgBr_2$ heated at 550 °C for 12 h, compared with the powder after washing with water.

In contrast, synchrotron PXRD measurements show the presence of $Mg(OH)_2$, indicating that $MgZrN_2$ is moisture sensitive (Figure S2). However, this synchtrotron PXRD measurement was

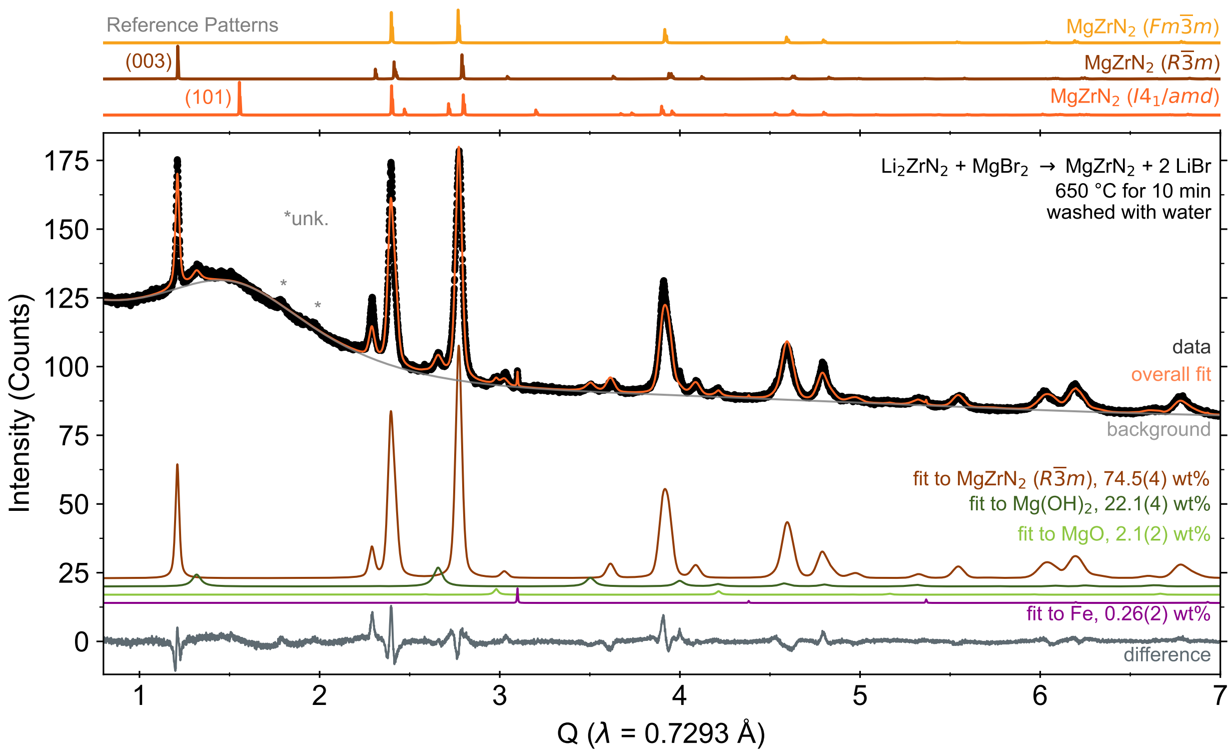

Figure S2: Synchrotron PXRD pattern of washed $R\bar{3}m$ $MgZrN_2$. Simulated patterns for $MgZrN_2$ polymorphs are shown for reference: $I4_1/amd$ (mp-1245429), $Fm\bar{3}m$ and $R\bar{3}m$ (this work). The trace Fe was likely contamination from a metal tool and does not appear in the laboratory PXRD pattern (Figure 1).

performed after this sample had experienced approximately two months of air exposure, while the laboratory PXRD measurement shown in Figure 1 were taken immediately after washing and drying the sample. It is unclear if the $Mg(OH)_2$ was present in the sample immediately after washing, or if it formed slowly over the two months of air exposure.

EDS measurements show a slight excess of Mg relative to Zr (Tables S1 and S2). Some of this Mg may be bound as MgO or $Mg(OH)_2$, as some Mg and O appear in the same part of the EDX map, in an area with no Zr (Figure S3, bottom left corner of images). A small amount of Br suggests the LiBr was not fully washed away (Li is not detectable by EDX) and the trace Hf is likely an impurity in the commercial ZrN precursor.

EDX also shows a substantially smaller amount of N than expected (Table S2). However, N and O quantification is unreliable via EDX as the emitted X-rays are low energy (compared to Mg and Zr), and therefore their quantification is more surface-sensitive. The measured oxygen content (56.5 at%) is substantially higher than would be expected even for a pure oxide, and likely comes from organic compounds (see carbon content) or other contamination. This measurement therefore suggests that the washed surface of this material is likely an oxynitride, but cannot assess if the bulk powder is a nitride or an oxynitride. To better confirm anion content in future experiments, combustion analysis should be performed on samples that are stored and washed under inert conditions (i.e., with anhydrous methanol).[1–3]

Table S1: Average atomic % of elements detected by EDX

Element	Average (at%)	St.Dev. (at%)
Mg	13.4	0.3
Zr	10.7	0.2
Hf	0.1	<0.1
N	8.0	1.4
O	56.5	0.9
C	11.2	0.2
Br	0.1	<0.1
Total	100.0	

Table S2: Key atomic ratios determined by EDX.

Ratios	Measured	Expected
Mg/(Mg+Zr)	0.56	0.5
N/(Mg+Zr)	0.33	1
N/(N+O)	0.12	1

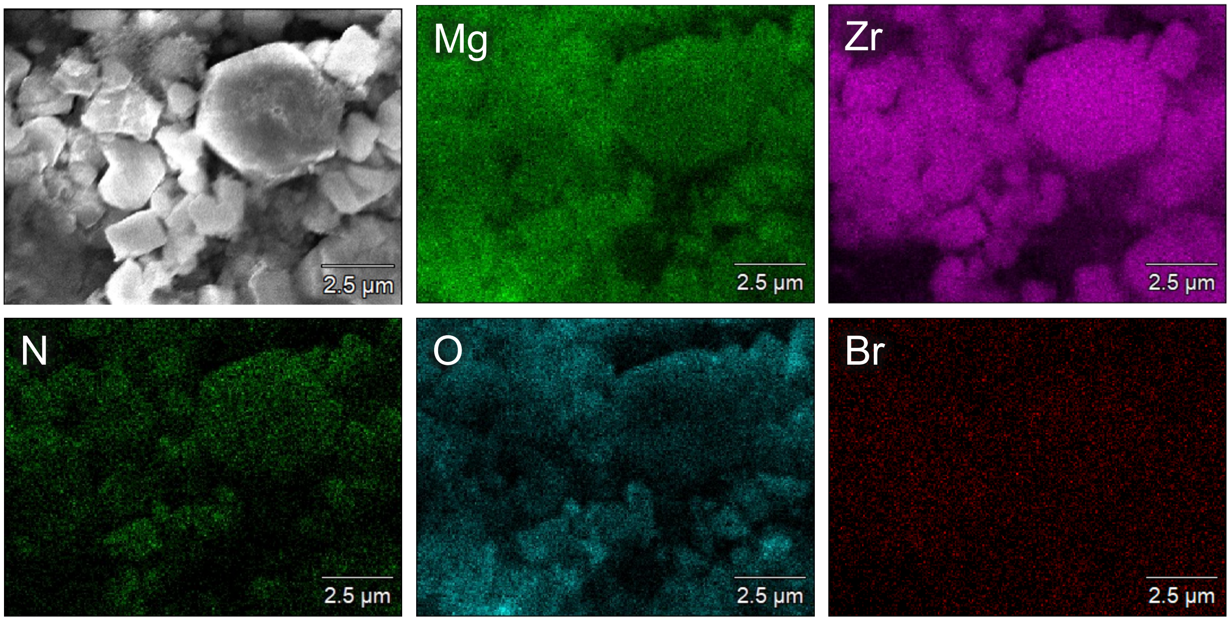

Figure S3: EDX mapping of $MgZrN_2$ particles after washing. The PXRD pattern for this sample is shown in Figure S2.

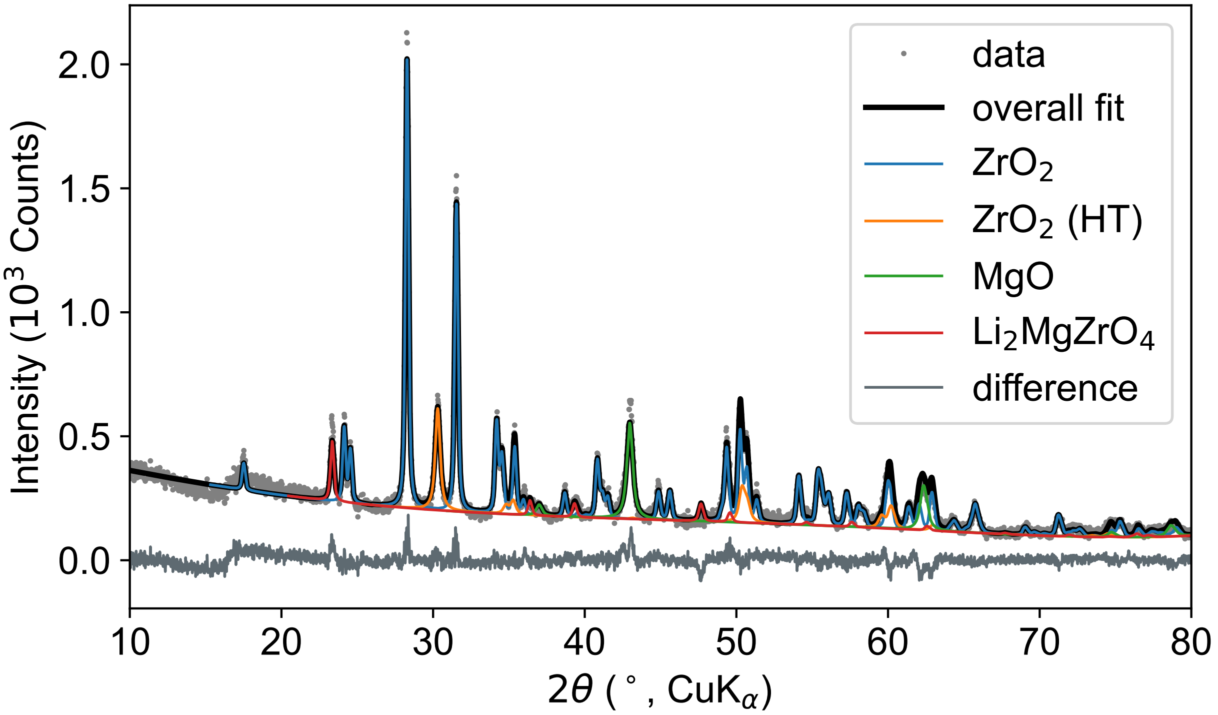

Figure S4: PXRD of the sample after heating $MgZrN_2$ under flowing O_2 at 900 °C.

Table S3: Summary of reaction products of $MgZrN_2$ heated under flowing O_2 to 900 °C for 10 min, with wt% determined via Rietveld analysis (Figure S4).

Phase	Spacegroup	PDF Card	wt%
ZrO_2	P21/c	01-083-0937	52.5
ZrO_2 (HT)	P42/nmc	01-070-7300	9.5
MgO	Fm-3m	00-045-0946	34.3
Li_2MgZrO_4	I41/amd	00-036-0308	3.6

Table S4: Atomic % of metals calculated from the quantitative phase analysis shown in Table S3.

	at% (metals basis)	ratio v. Zr
Li	1.3	0.04
Mg	61.7	1.67
Zr	37.0	1.00

To assess the possibility of residual Li in our sample, we oxidized an aliquot of the washed $MgZrN_2$ (Figure 1) at 900 °C under flowing oxygen (100 sccm). The resulting PXRD pattern shows a Li-containing material, Li_2MgZrO_4, along with MgO, monoclinic ZrO_2, and a high temperature (HT) tetragonal polymorph of ZrO_2 (Figure S4). Quantitative phase analysis via the Rietveld method indicates that Li_2MgZrO_4 comprises 3.6 wt% of the total sample after oxidation (Table S3). This weight fraction corresponds to 1.3 at% Li on a metals basis for the sample (Table S4), indicating only trace Li remains after washing. We note that the Mg/(Mg+Zr) ratio determined from this PXRD analysis is higher than the ratio detected by EDX on the same sample prior to oxidation (0.62 for PXRD v. 0.56 for EDX). However, the Li_2MgZrO_4 phase exhibits cation disorder on the Mg/Zr site and may be Mg-deficient, but we did not refine cation occupancies. In sum, this result shows that a small amount of Li is present in the washed sample.

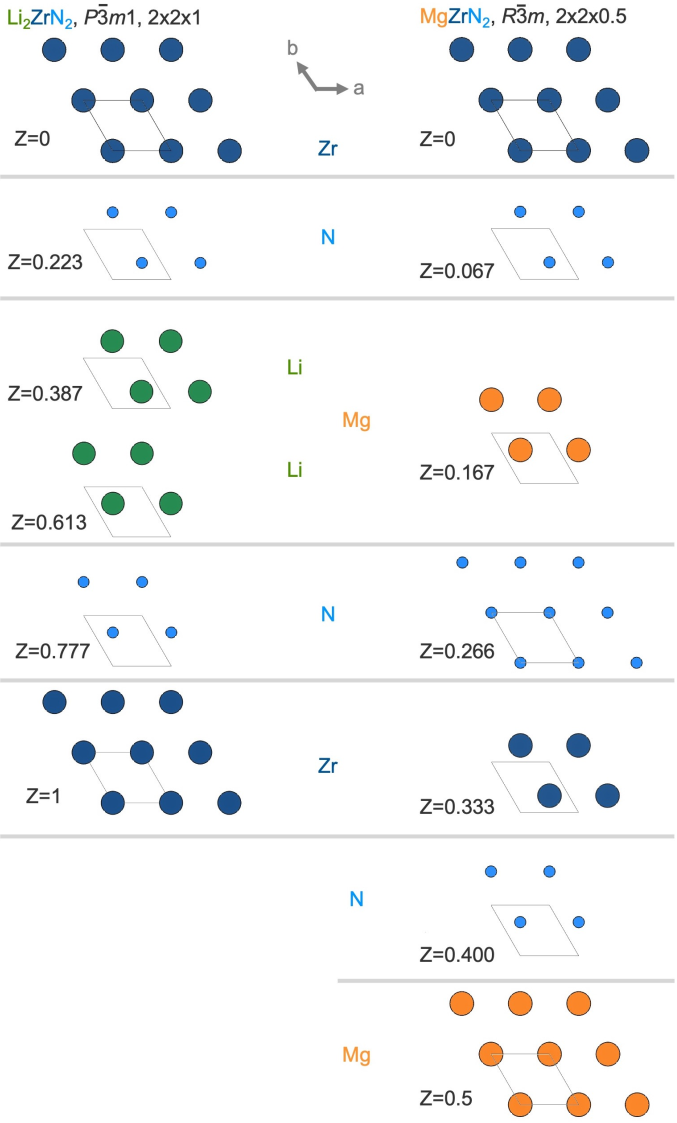

Figure S5: Visual comparison of the stacking sequence for Li_2ZrN_2 (left, 2x2x1 supercell) and $MgZrN_2$ (right, 2x2x0.5 supercell).

This ion exchange synthesis is topotactic. The Zr-layers are retained from the Li_2ZrN_2 precursor to the $MgZrN_2$ product in the α-$NaFeO_2$ structure type. However, this transformation involves a shift of the Zr-containing layers and the anion sublattice. Figure S5 shows that Li_2ZrN_2 exhibits hexagonal-close-packing for the anion sublattice and cubic-close-packing for the cation sublattice, with Li in the tetrahedral voids between the anion layers. The cation sublattice of $MgZrN_2$ is also cubic-close-packed, but the anion sublattice shifts from hexagonal- to cubic-close-packed.

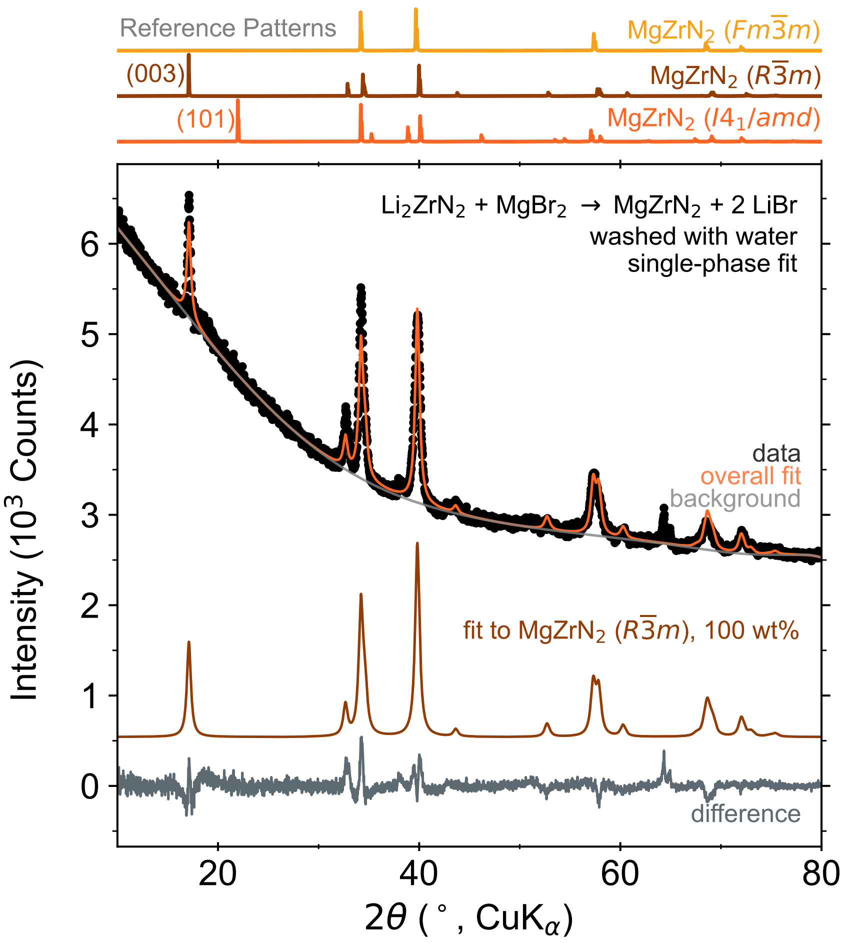

Figure S6: The same PXRD data as shown in Figure 1, but fit with a single $R\bar{3}m$ structure with refined Mg and Zr site disorder.

Refining anti-site defects indicates a substantial amount of site disorder. The refinement shown in Figure S6 shows 16% anti-site defect concentrations (i.e., $(Mg_{0.84}Zr_{0.16})(Zr_{0.84}Mg_{0.16})N_2$). This fit has a higher R_{wp} value than the two-phase fit shown in Figure 1 (4.709% and 4.336%, respectively). A similar single-phase fit using synchrotron PXRD data showed 19% anti-site defects (Figure S2).

Reaction temperature and dwell time influences relative amounts of $R\bar{3}m$ and $Fm\bar{3}m$ $MgZrN_2$ present in the sample. Higher temperatures and longer heating times tend to increase the fraction of the cation-disordered phase ($Fm\bar{3}m$). For example, identical reaction mixtures heated to 650 °C for a 10 min dwell time (Figure 1) and a 12 h dwell time (Figure S7) showed different phase

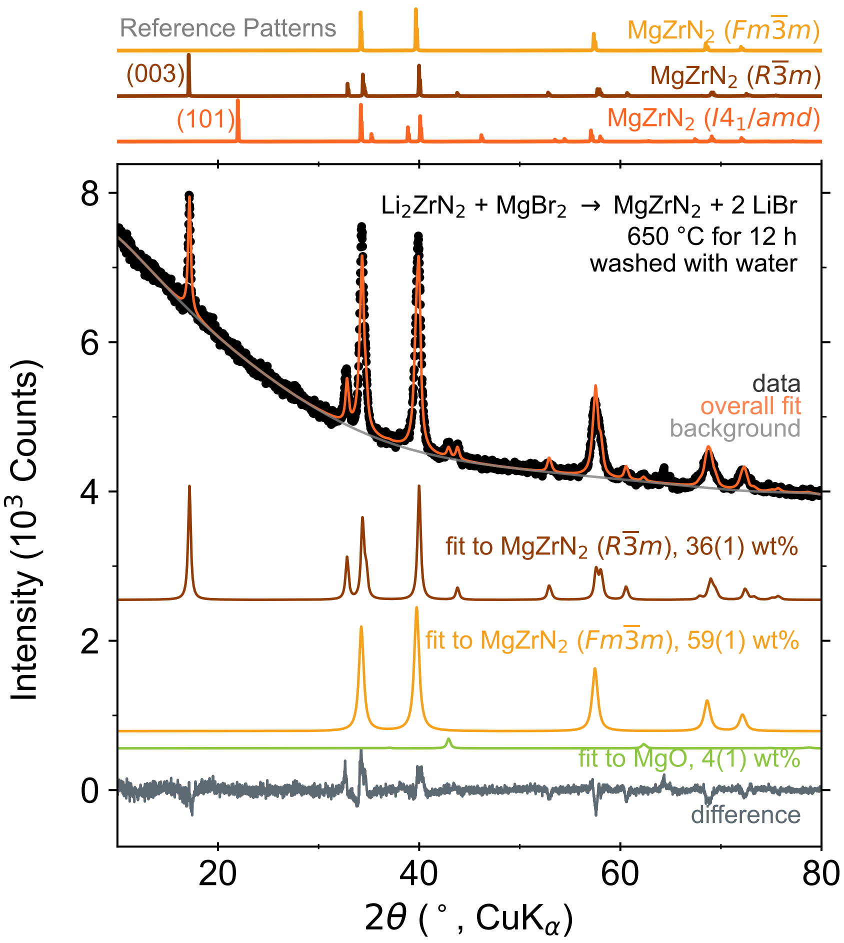

Figure S7: PXRD of the washed reaction products for $Li_2ZrN_2 + MgBr_2$ heated at 650 °C for 12 h.

Table S5: Summary of weight percents of reaction products between $Li_2ZrN_2 + MgBr_2$ heated to 650 °C for different dwell times. Phase fractions determined by Rietveld analysis, with fits shown in the associated figures.

Dwell time	$MgZrN_2$ ($R\bar{3}m$)	$MgZrN_2$ ($Fm\bar{3}m$)	MgO	Fit
10 min	47(1) wt%	53(1) wt%	-	Figure 1
12 h	36(1) wt%	59(1) wt%	4(1) wt%	Figure S7

fractions (Table S5). These differing structures also lead to different optical properties (Figure S8). While we did not optimize the synthesis conditions for phase-pure $R\bar{3}m$ $MgZrN_2$ here, this finding suggests that cooler reaction temperatures and shorter reactions times would be preferable.

Additional UV-vis experiments on $MgZrN_2$

The reaction products appear reddish, although this color becomes darker with higher reaction temperatures or longer reaction times. Figure S8 compares the spectra of two samples of $MgZrN_2$ reacted at 650 °C for either 10 min or 12 h. The powder from the longer reaction (black trace) looks black and absorbs more light at low photon energy compared to the 10 min reaction. In contrast, the 10 min reaction shows a sharper absorption onset near 2 eV, consistent with the calculated absorption onset for $R\bar{3}m$ $MgZrN_2$. The 10 min reaction was also presented in the main text (Figure 3).

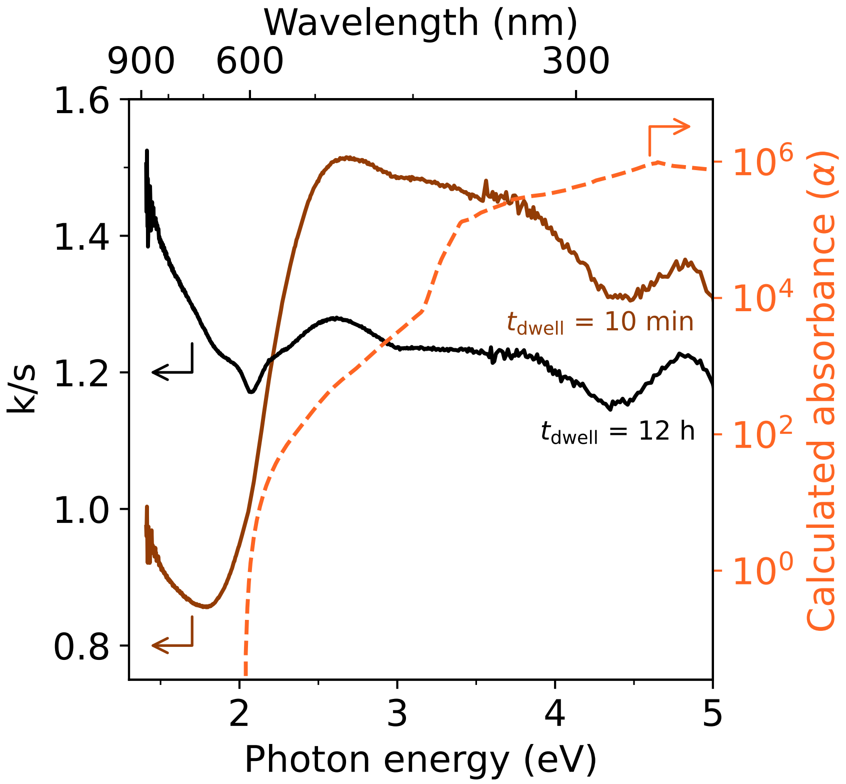

Figure S8: UV-vis spectra for the washed powders of $MgZrN_2$ prepared from the reaction between $Li_2ZrN_2 + MgBr_2$ heated to 650 °C for two different dwell times: 10 min (brown trace) and 12 h (black trace). For reference, the calculated absorbance is also shown (dashed line).

Ensemble calculations for $MgZrN_2$

We computed relaxed total energies for all of the different configurations of $MgZrN_2$. Symmetry inequivalent cation occupations and their degeneracies were identified using AFLOW.[4,5] The probabilities of each configuration at different temperatures were then calculated as $p_i = (1/Z)g_i * exp(-\Delta E_i/kT)$, where $Z = \sum_i p_i$ is the partition function, g_i is the degeneracy of the configuration, and ΔE_i is the energy of the configuration above the ground state. Diffraction patterns were generated for each configuration, and are ensemble averaged with the weights p_i. The γ-$LiFeO_2$ structure is the lowest energy structure, as expected, so the low temperature ensemble pattern is heavily weighted towards this polymorph ($T = 101$ K). The (101) reflection—which indicates cation order for this polymorph—decays rapidly as the equilibrium temperature increases (Figure S9). The thermodynamics of these configurations suggest $MgZrN_2$ should exhibit disorder at equilibrium, even at relatively low temperatures (e.g., 501 K). This supports the idea that synthesized α-$NaFeO_2$-type $MgZrN_2$ ($R\bar{3}m$) is kinetically trapped in the ordered structure.

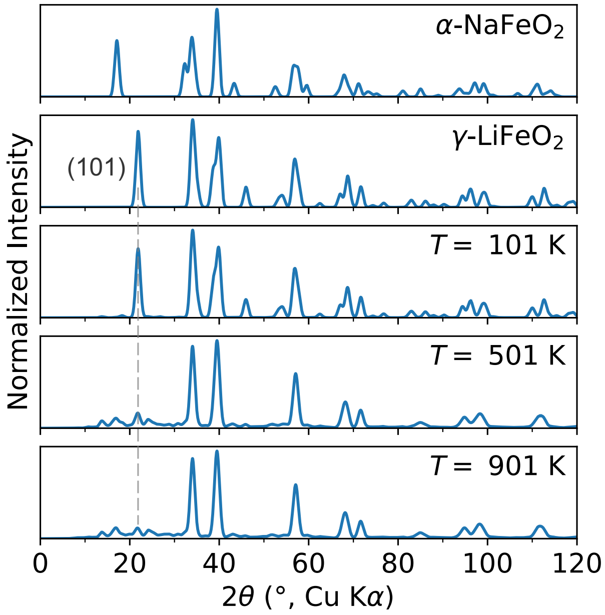

Figure S9: Simulated diffraction patterns $MgZrN_2$ in the α-$NaFeO_2$ and the γ-$LiFeO_2$ polymorphs, along with simulated patterns for the global minimum of Gibbs free energy at zero pressure and given temperatures, calculated from the ensemble average.

In situ synchrotron PXRD experiments

Figure S10 shows a larger version of the heatmap presented in Figure 4, along with select fits conducted as part of the Rietveld analysis.

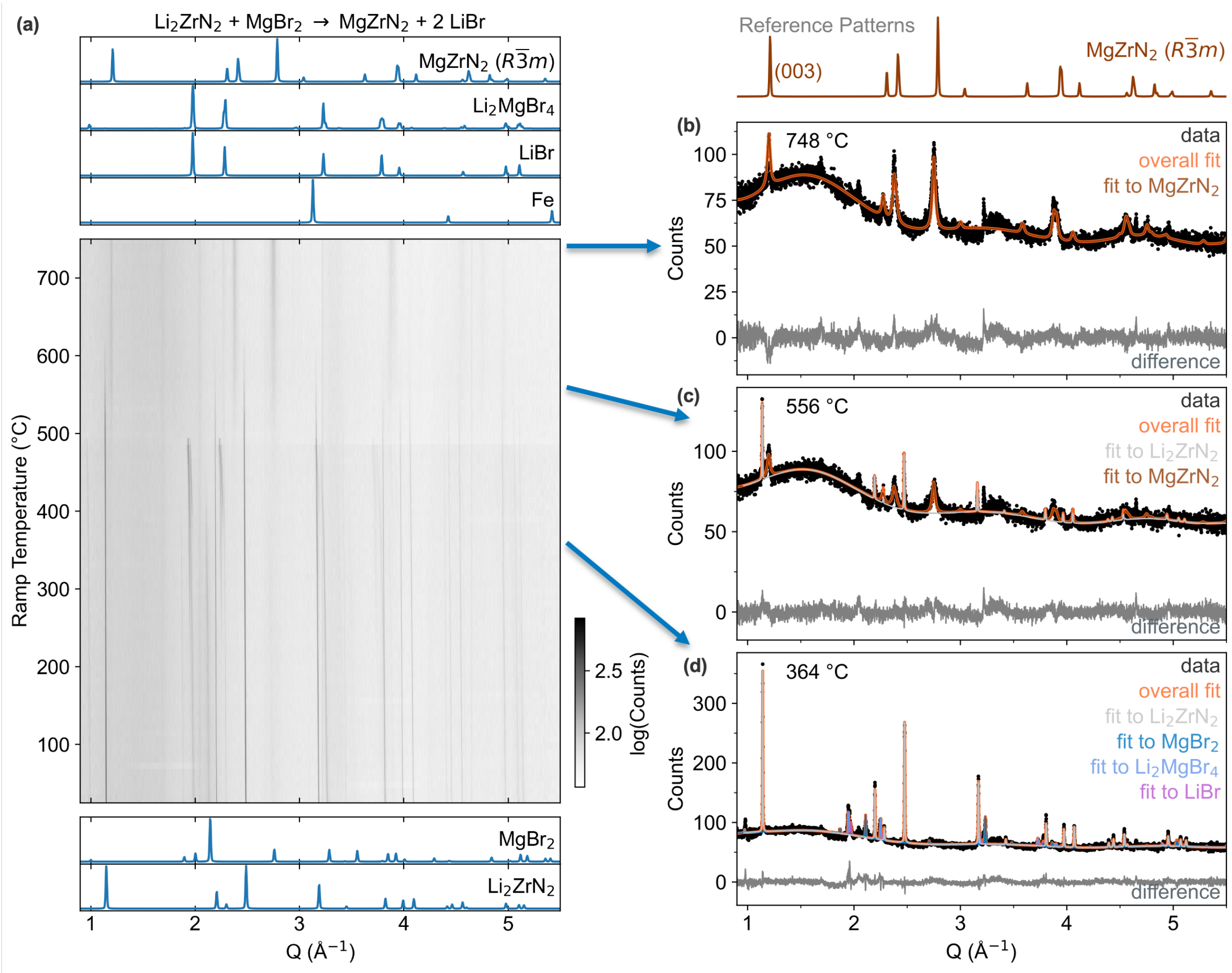

Figure S10: *In situ* synchrotron PXRD of $Li_2ZrN_2 + MgBr_2$ heated at +10°C/min showing (a) all patterns as a heatmap and select patterns at (b) 364 °C, (c) 556 °C, and (d) 748 °C fit via Rietveld analysis.

In situ synchrotron PXRD experiments were conducted in attempts to synthesize layered $R\bar{3}m$ structures for $FeZrN_2$ (Figure S11), $CuZrN_2$ (Figure S12), and $ZnZrN_2$ (Figure S13). Strong reflections are observed for metallic Fe, Cu, and Zn, along with rocksalt ZrN, beginning as low as 100 °C in the case of Fe. These reduced metals indicate the loss of nitrogen gas from the structure ($3A^{2+} + 2N^{3-} \longrightarrow 3A + N_2$), and the rapid decomposition of any AZrN$_2$ (A = Fe, Cu, Zn) that

may be forming. This gas release caused the Fe-containing capillary to pop at approximately 400 °C, as seen by the sharp change in the diffraction. The loss of nitrogen from the solid suggests that $FeZrN_2$, $CuZrN_2$, and $ZnZrN_2$ are not stable at the temperatures necessary for solid-state diffusion between Li_2ZrN_2 and the precursor salts (≥200 °C). Alternatively, self-heating from the exothermic ion exchange reaction may drive local temperatures above the decomposition point of the ternary nitrides.

We attempted to mitigate self-heating for the Zn-based reaction via the addition of a LiCl/KCl eutectic flux (50 wt%), but decomposition was still observed (Figure S13). Metallic Zn appears in the diffraction patterns near 200 °C, indicating decomposition. There are small peaks that are we were unable to index, including a small peak near Q = 1.2 $Å^{-1}$ that aligns well with the supercell reflections for the possible $ZnZrN_2$ polymorphs: (003) for the $R\bar{3}m$ structure, or (001) for the $P3m1$ structure (Figure S13b-d). However, our attempts to fit these patterns with these phases were unsuccessful. Interestingly, ball-milling the $Li_2ZrN_2 + ZnBr_2$ with the LiCl/KCl flux resulted in apparent reactivity between the KCl and the $ZnBr_2$, as the $ZnBr_2$ phase is not visible in the initial diffraction pattern (Figure S13d). Instead, broad peaks that index to a rocksalt phase–which we fit as K(Cl,Br) are present. This phase likely contains the Zn, but we were unable to refine the metal site occupancy owing to the similar scattering powers of K and Zn. Reactivity between $ZnBr_2$ and KCl is not to blame for the formation of Zn metal; *ex situ* experiments without a LiCl/KCl flux also yielded metallic Zn as a decomposition product, even with low reaction temperatures (Figures S14, S15). Further *in situ* synchrotron PXRD experiments with higher energy and higher flux X-rays are needed to better understand this reaction process.

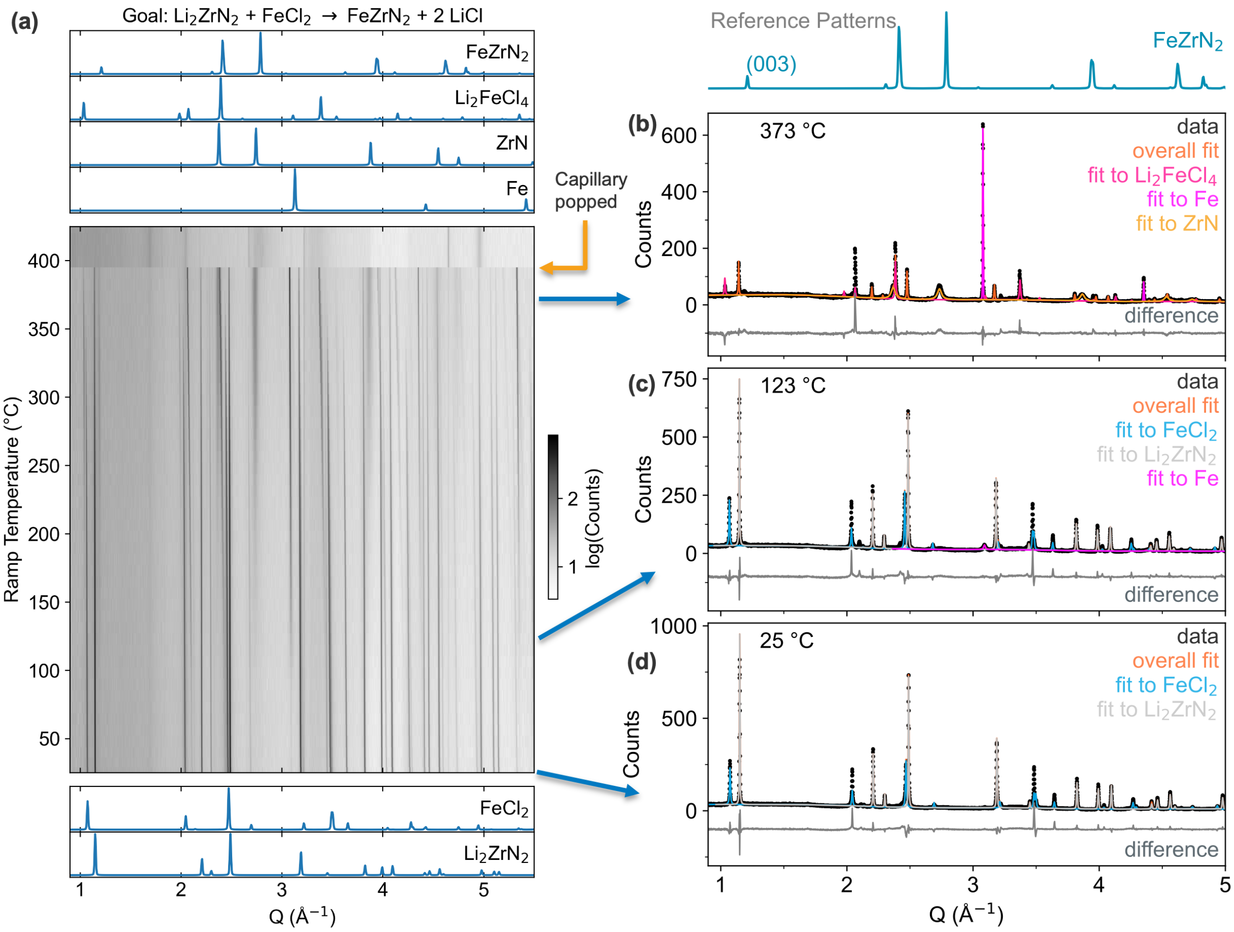

Figure S11: *In situ* synchrotron PXRD of $Li_2ZrN_2 + FeCl_2$ heated at +10°C/min showing (a) all patterns as a heatmap and select patterns at (b) 373 °C, (c) 123 °C, and (d) 25 °C fit via Rietveld analysis. The $FeZrN_2$ reference pattern was generated with VESTA[6] by replacing Mg in the $R\bar{3}m$ $MgZrN_2$ structure with Fe.

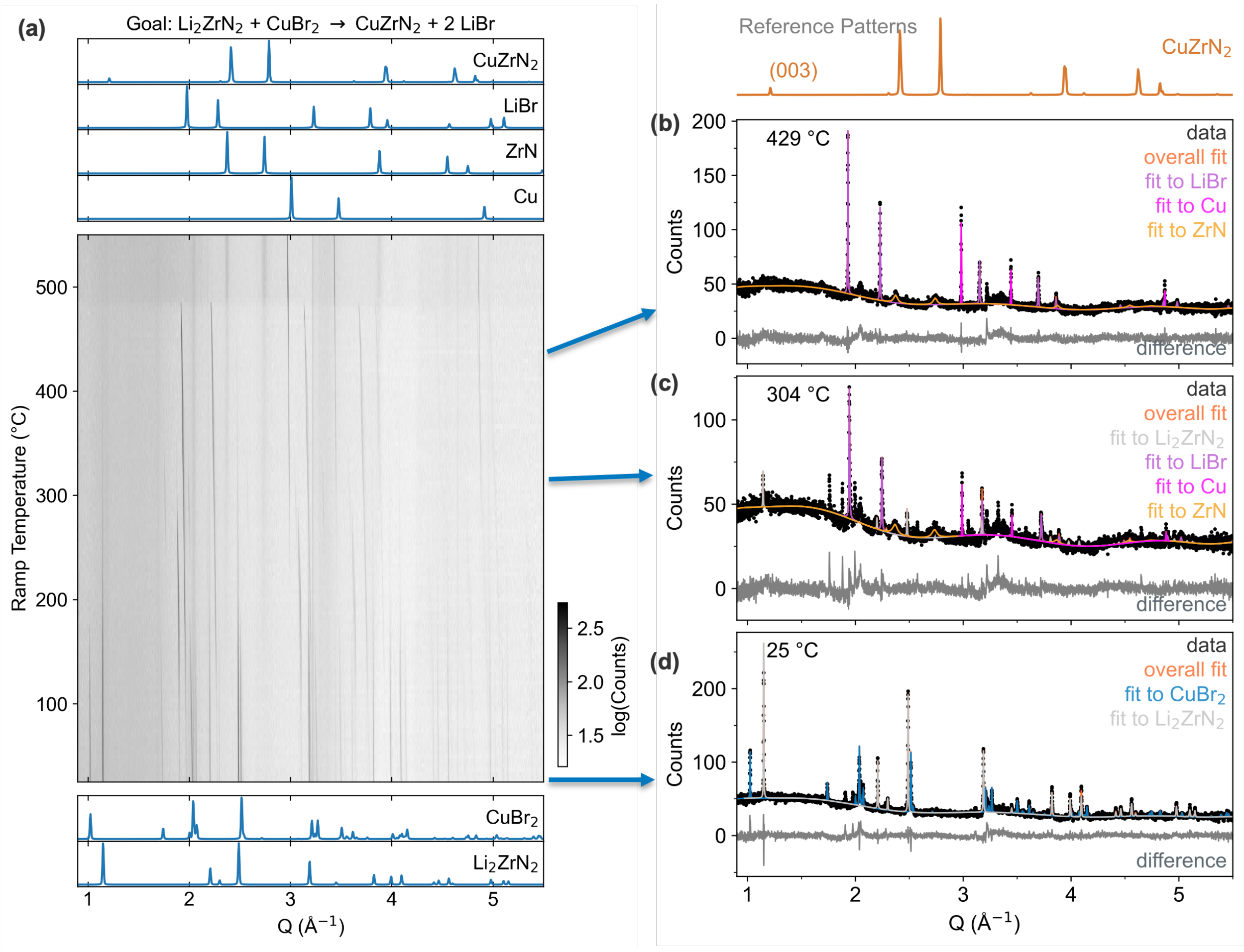

Figure S12: *In situ* synchrotron PXRD of $Li_2ZrN_2 + CuBr_2$ heated at +10°C/min showing (a) all patterns as a heatmap and select patterns at (b) 429 °C, (c) 304 °C, and (d) 25 °C fit via Rietveld analysis. The $CuZrN_2$ reference pattern was generated with VESTA[6] by replacing Mg in the $R\bar{3}m$ $MgZrN_2$ structure with Cu.

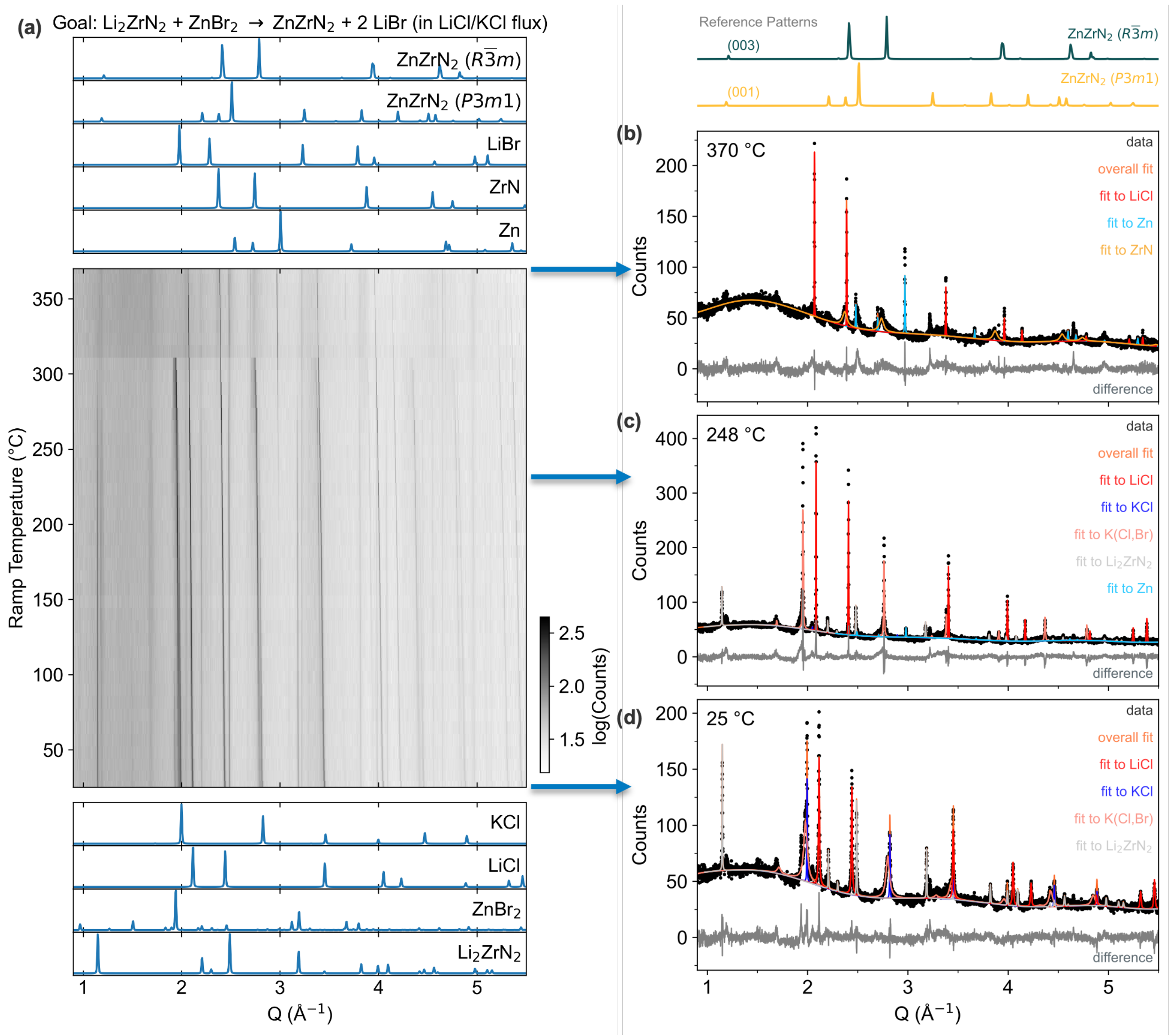

Figure S13: *In situ* synchrotron PXRD of $Li_2ZrN_2 + ZnBr_2$ (ball-milled with LiCl/KCl eutectic as a heat sink) heated at +10°C/min showing (a) all patterns as a heatmap and select patterns at (b) 370 °C, (c) 248 °C, and (d) 25 °C fit via Rietveld analysis. The $ZnZrN_2$ $R\bar{3}m$ reference pattern was generated with VESTA[6] by replacing Mg in the $R\bar{3}m$ $MgZrN_2$ structure with Fe. The $P3m1$ pattern was generated from mp-1014244.

Ex situ synthetic attempts towards $ZnZrN_2$

Ex situ PXRD of reactions between $Li_2ZrN_2 + ZnBr_2$ do not show evidence of $ZnZrN_2$ (Figures S14 and S15). Instead, decomposition products are detected ($ZnZrN_2 \longrightarrow Zn + ZrN + \frac{1}{6}N_2$). The reaction held at 200 °C proceeded only partially (Figure S14), yielding $ZrN + Zn$ along with unreacted Li_2ZrN_2 and an intermediate Li_2ZnBr_4. The Li_2ZnBr_4 forms via the combination of the LiBr product and the $ZnBr_2$ precursor. At 300 °C, the reaction proceeds completely within 10 h (Figure S15), with the PXRD showing only $Zn + ZrN + 2LiBr$.

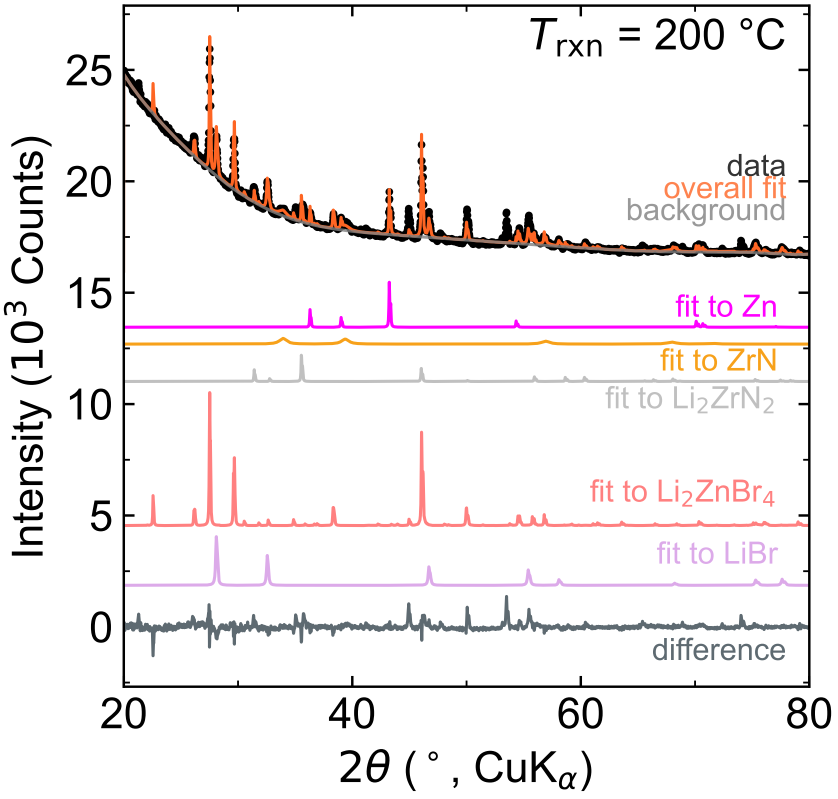

Figure S14: PXRD of the reaction products for $Li_2ZrN_2 + ZnBr_2$ heated at 200 °C for 10 h.

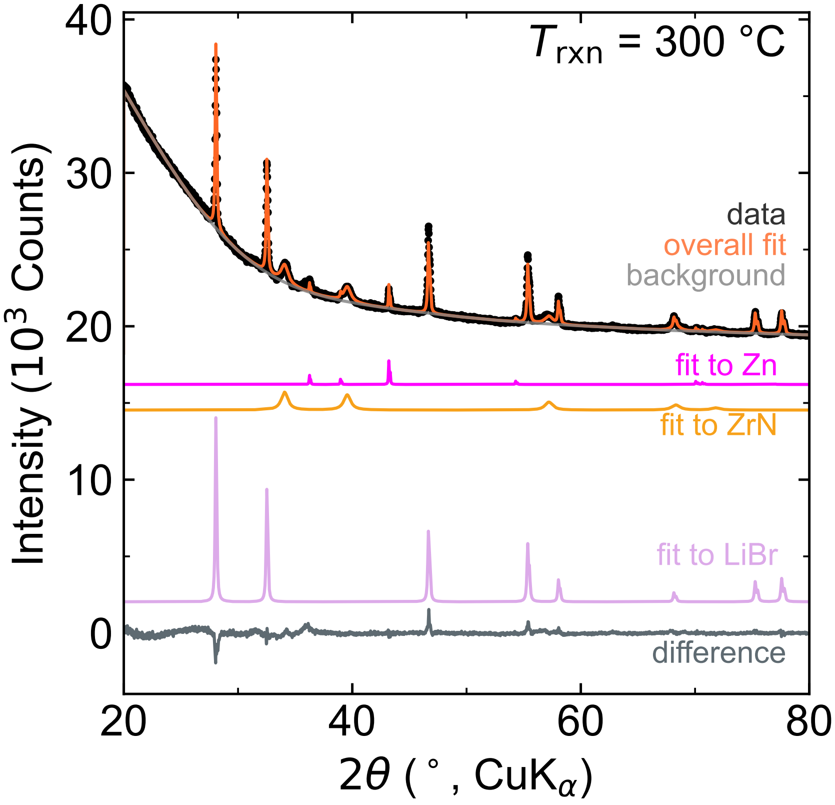

Figure S15: PXRD of the reaction products for $Li_2ZrN_2 + ZnBr_2$ heated at 300 °C for 10 h.

Ion exchange thermodynamics

Temperature dependent free energy calculations for ion exchange reactions targeting α-$NaFeO_2$-type $ZnZrN_2$ and $MgZrN_2$ are shown in Figure S16. While ion exchange targeting $MgZrN_2$ is more exergonic than the reaction targeting $ZnZrN_2$, both reactions have a substantial forward driving force. Therefore, the fact that $MgZrN_2$ was synthesized while $ZnZrN_2$ was not suggests that a different thermodynamic or kinetic factor matters.

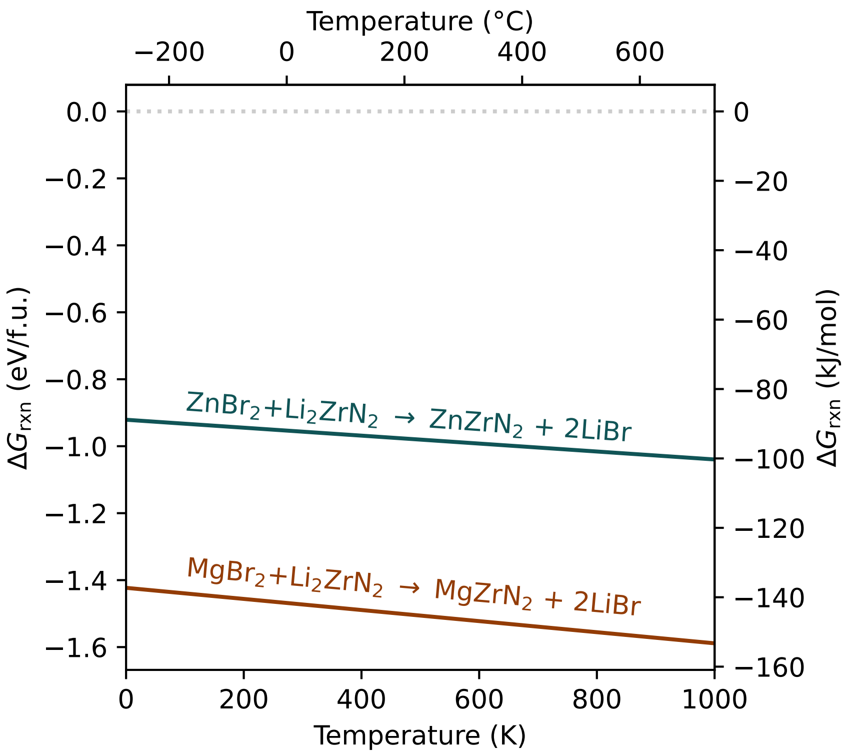

Figure S16: Overall $\Delta G_{\text{rxn}}(T)$ energies for the reactions targeting $ZnZrN_2$ and $MgZrN_2$ beginning from bromide salts.

Hess's law for reaction enthalpies allows us to consider the nitride and halide parts of the ion exchange reaction independently. Consider the general reaction of interest here:

$$\text{Li}_2\text{ZrN}_2 + AX_2 \longrightarrow A\text{ZrN}_2 + 2\text{Li}X$$

By Hess's law,

$$\Delta H_{\text{rxn}} = (\text{products}) - (\text{reactants}) = (2 * \Delta H_{\text{f,Li}X} + \Delta H_{\text{f,}A\text{ZrN2}}) - (\Delta H_{\text{f,Li2ZrN2}} + \Delta H_{\text{f,}AX2})$$

For a given target compound $AZrN_2$, the components $\Delta H_{\text{Li2ZrN2}}$ and ΔH_{AZrN2} can be treated as a constant because they cannot be changed by experimental choices. However, an experimenter can pick which halide X to use for the reaction based on factors like melting point, solubility, and reaction enthalpy. To evaluate the thermodynamics of this choice, the overall Hess's law equation for the reaction can be simplified to

$$(2 * \Delta H_{\text{f,Li}X}) - (\Delta H_{\text{f,}AX2})$$

Which is equivalent to the reaction $2\text{Li} + AX_2 \longrightarrow 2\text{Li}X + A$. Generally, these values are substantially negative (LiX halides are extremely stable), which means reactions tend to proceed forwards. Figure S17 shows how choice of X affects the ion exchange reaction energetics for the ion exchanges reactions presented here. Notably, reactions beginning from transition metal halides are substantially more exothermic than the MgX_2 ion exchanges, owing to the less negative $\Delta H_{\text{f,}AX2}$ values for A = Fe, Cu, Zn compared to Mg.

In contrast, if the goal is just to consider stability of the $AZrN_2$ phase, then overall reaction enthalpy is less relevant compared to the energy of the competing phases (e.g., elements and binaries). Therefore, we created Figure 5 to show the relative stability of $AZrN_2$ phases against decomposition.

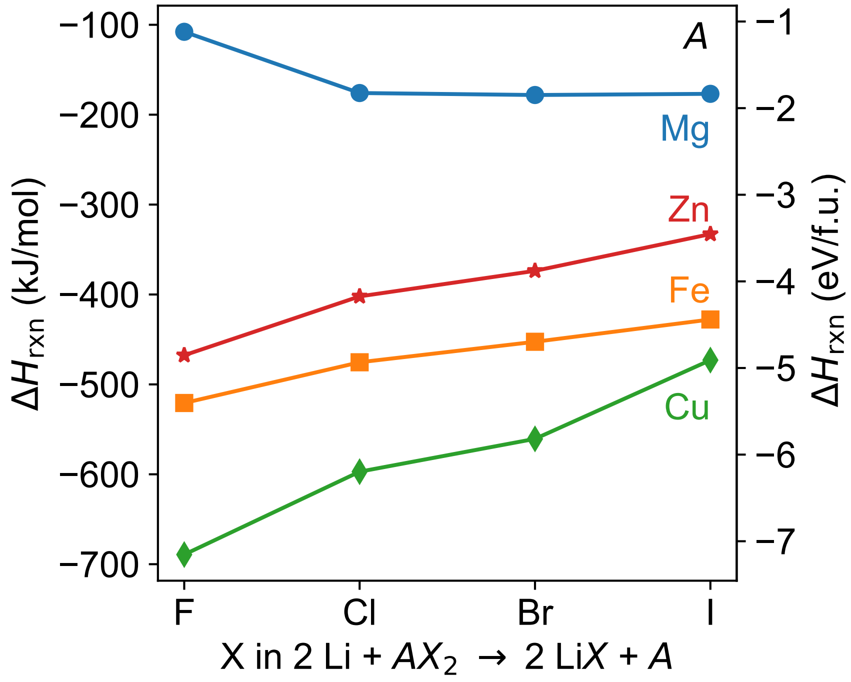

Figure S17: Reaction enthalpies calculated for $2\mathrm{Li} + AX_2 \longrightarrow 2\mathrm{Li}X + A$ using ΔH_f values from the CRC Handbook of Chemistry and Physics.[7]

Li-M-N entries in the PDF 2021 database

The data used for coloring the periodic table shown in Figure 6 were collected by searching the Powder Diffraction File (PDF) database using the following criteria:

- Element filter = Yes for Li, N
- Element filter = Maybe for all metals and metalloids.
- Number of elements = 3

These data are presented in Table S6.

Table S6: Li-M-N ternaries reported in the PDF 2021 database

PDF #	Empirical formula	Metal	SpaceGroup
00-007-0245	Al Li3 N2	Al	206
01-074-0142	Al Li3 N2	Al	206
01-086-3966	Al Li3 N2	Al	206
03-065-3189	Al Li3 N2	Al	206
01-074-1358	B Li3 N2	B	14
01-075-3054	B Li3 N2	B	14
00-040-1166	B Li3 N2	B	94
01-075-3053	B Li3 N2	B	94
01-080-2274	B Li3 N2	B	136
01-075-3051	B Li3 N2	B	141
01-075-3052	B Li3 N2	B	141
00-013-0393	B Li3 N2	B	
00-016-0273	B Li3 N2	B	
00-050-1495	B Li3 N2	B	
00-023-1175	Ba Li N	Ba	

01-077-3911	Ba2 Li N	Ba	137
01-077-3912	Ba3 Li N	Ba	194
00-059-0159	Ba3 Li N	Ba	194
01-086-1793	Be Li N	Be	14
01-072-9363	Ca Li N	Ca	62
00-018-0724	Ca Li N	Ca	
01-083-8888	Ca3 Li2 N6	Ca	51
00-024-0603	Ce Li2 N2	Ce	164
01-076-0449	Ce Li2 N2	Ce	164
00-005-0605	Co0.43 Li2.57 N	Co	191
03-065-9918	Co0.43 Li2.57 N	Co	191
01-071-7305	Co0.46 Li2.54 N	Co	191
01-073-3896	Co0.53 Li1.99 N	Co	191
01-085-4405	Co0.43 Li2.57 N	Co	191
00-036-0695	Cr Li9 N5	Cr	
01-089-2897	Cr0.8 Li7.2 N4	Cr	225
01-079-1124	Cr2 Li15 N9	Cr	130
01-074-3506	Cu0.06 Li2.94 N	Cu	191
01-080-5162	Cu0.167 Li2.833 N	Cu	191
01-080-5163	Cu0.289 Li2.711 N	Cu	191
01-085-4412	Cu0.3 Li2.7 N	Cu	191
03-065-9949	Cu0.3 Li2.7 N	Cu	191
01-080-5164	Cu0.348 Li2.652 N	Cu	191
01-070-3089	Cu0.39 Li2.6 N	Cu	191
01-070-7316	Cu0.404 Li2.576 N	Cu	191
01-070-7315	Cu0.423 Li2.558 N	Cu	191
01-070-7313	Cu0.428 Li2.572 N	Cu	191

01-072-6104	Cu0.43 Li2.25 N	Cu	191
01-070-7314	Cu0.43 Li2.57 N	Cu	191
01-074-3507	Cu0.43 Li2.57 N	Cu	191
01-073-7690	Cu2.81 Li0.1 N	Cu	221
01-073-7691	Cu2.88 Li0.13 N	Cu	221
01-073-7695	Cu2.94 Li0.85 N	Cu	221
01-073-7696	Cu2.94 Li1.03 N	Cu	221
01-073-7692	Cu3 Li0.42 N	Cu	221
01-073-7693	Cu3 Li0.52 N	Cu	221
01-073-7694	Cu3 Li0.64 N	Cu	221
01-080-0718	Fe Li3 N2	Fe	72
00-020-0626	Fe Li3 N2	Fe	
01-080-0888	Fe Li4 N2	Fe	71
01-083-6626	Fe0.26 Li2.74 N	Fe	191
01-089-1762	Fe0.63 Li2.37 N	Fe	191
01-073-9746	Fe0.86 Li3 N2	Fe	72
00-055-0451	Ga Li3 N2	Ga	206
01-074-0143	Ga Li3 N2	Ga	206
01-077-6079	Ga Li3 N2	Ga	206
03-065-3190	Ga Li3 N2	Ga	206
00-028-0566	Ge Li N	Ge	
00-028-0565	Ge Li2 N2	Ge	
00-027-1240	Ge Li8 N4	Ge	206
01-082-6921	Ge0.67 Li3.33 N2	Ge	206
01-074-0161	Ge10.67 Li53.33 N32	Ge	206
00-027-1239	Ge2 Li N3	Ge	36
01-082-6922	Ge2 Li N3	Ge	36

01-086-6831	Ge2 Li N3	Ge	36
00-027-0293	Hf Li2 N2	Hf	164
00-049-1181	In Li3 N2	In	
01-070-7485	Li Mg N	Mg	62
01-079-5487	Li Mg N	Mg	62
01-079-5488	Li Mg N	Mg	62
01-079-5489	Li Mg N	Mg	62
00-006-0702	Li Mg N	Mg	225
01-072-1287	Li Mg N	Mg	225
01-079-5490	Li Mg N	Mg	225
01-079-5491	Li0.24 Mg2.76 N1.838	Mg	199
01-079-5493	Li0.24 Mg2.76 N1.92	Mg	199
01-079-5494	Li0.24 Mg2.76 N1.92	Mg	199
01-079-5495	Li0.24 Mg2.76 N1.92	Mg	199
01-079-5492	Li0.48 Mg2.52 N1.84	Mg	199
01-070-7484	Li0.51 Mg2.49 N1.83	Mg	199
01-079-5500	Li1.09 Mg0.91 N0.97	Mg	62
01-079-5497	Li1.10 Mg0.90 N0.96	Mg	225
01-079-5499	Li1.10 Mg0.90 N0.96	Mg	225
01-079-5496	Li1.11 Mg0.89 N0.96	Mg	225
01-079-5498	Li1.11 Mg0.89 N0.96	Mg	225
01-070-7486	Li1.12 Mg0.88 N0.96	Mg	225
01-089-1030	Li0.66 Mn1.34 N	Mn	136
01-089-1031	Li0.86 Mn1.14 N	Mn	136
01-089-1764	Li2.27 Mn0.73 N	Mn	191
01-070-9584	Li2.33 Mn0.67 N	Mn	191
01-070-9582	Li24 Mn3 N10.86	Mn	163

01-070-9583	Li6.23 Mn1.77 N3	Mn	189
01-074-9559	Li7 Mn N4	Mn	218
00-051-1219	Li7 Mn N4	Mn	218
01-070-9581	Li7 Mn N4	Mn	218
01-089-4135	Li7 Mn N4	Mn	218
01-081-0578	Li Mo N2	Mo	160
00-047-1478	Li Mo N2	Mo	166
01-079-1122	Li6 Mo N4	Mo	137
01-070-6781	Li N Na2	Na	191
01-070-6787	Li N2 Na5	Na	1
01-070-6786	Li N2 Na5	Na	5
01-070-6779	Li2 N Na	Na	191
01-070-6780	Li2 N2 Na4	Na	129
01-070-6783	Li3 N2 Na3	Na	6
01-070-6782	Li3 N2 Na3	Na	115
01-070-6778	Li4 N2 Na2	Na	65
01-070-6776	Li4 N2 Na2	Na	139
01-070-6777	Li4 N2 Na2	Na	225
01-070-6785	Li5 N2 Na	Na	6
01-070-6784	Li5 N2 Na	Na	123
00-053-0436	Li N4 Nb3	Nb	193
01-081-0211	Li7 N4 Nb	Nb	205
01-078-6910	Li N Ni	Ni	187
01-089-6875	Li N Ni	Ni	187
01-072-6105	Li1.35 N Ni0.79	Ni	191
01-086-5609	Li2 N Ni0.67	Ni	191
01-086-5610	Li2 N Ni0.67	Ni	191

01-074-3503	Li2.15 N Ni0.85	Ni	191
01-071-9493	Li2.57 N Ni0.43	Ni	191
01-074-3505	Li2.63 N Ni0.37	Ni	191
01-074-3504	Li2.93 N Ni0.07	Ni	191
01-089-6876	Li5 N3 Ni3	Ni	189
01-086-2147	Li5.69 N3 Ni2.31	Ni	189
01-086-5611	Li5.69 N3 Ni2.31	Ni	189
03-065-3353	Li7 N4 Ni	Ni	218
01-070-6922	Li5 N4 Re	Re	59
01-071-6773	Li5 N4 Re	Re	59
01-072-8199	Li3 N2 Sc	Sc	206
00-026-1186	Li N3 Si2	Si	36
00-050-0747	Li N3 Si2	Si	36
01-070-3183	Li N3 Si2	Si	36
01-076-0517	Li N3 Si2	Si	36
01-086-6830	Li N3 Si2	Si	36
00-040-1447	Li18 N10 Si3	Si	
01-078-8859	Li2 N2 Si	Si	61
00-023-0365	Li2 N2 Si	Si	
00-040-1448	Li21 N11 Si3	Si	
00-007-0260	Li5 N3 Si	Si	206
00-040-1446	Li5 N3 Si	Si	225
01-074-0159	Li53.33 N32 Si10.67	Si	206
01-083-4019	Li54.88 N30.98 Si9.12	Si	79
00-040-1449	Li8 N4 Si	Si	
01-089-6184	Li N Sr	Sr	131
00-023-0367	Li N Sr	Sr	

01-089-6183	Li4 N2 Sr	Sr	141
01-081-0296	Li0.977 N4 Ta3.023	Ta	193
01-081-0295	Li0.983 N4 Ta3.017	Ta	193
01-081-0290	Li1.8 N4 Ta2.2	Ta	225
01-081-0289	Li1.85 N4 Ta2.15	Ta	225
01-071-3446	Li4 N3 Ta	Ta	73
01-079-2303	Li7 N4 Ta	Ta	205
01-078-7112	Li8 N2 Te	Te	109
01-078-7113	Li8 N2 Te	Te	109
00-025-0502	Li2 N2 Th	Th	147
01-072-0794	Li2 N2 Th	Th	147
00-006-0703	Li5 N3 Ti	Ti	206
01-074-0160	Li53.33 N32 Ti10.67	Ti	206
01-074-3519	Li N2 U	U	141
00-025-0504	Li2 N2 U	U	147
01-082-7245	Li2 N2 U	U	147
01-072-7053	Li7 N4 V	V	137
01-070-5088	Li7 N4 V	V	205
01-072-7052	Li7 N4 V	V	205
00-015-0216	Li7 N4 V	V	218
01-072-7051	Li7 N4 V	V	218
01-089-3703	Li7 N4 V	V	218
00-049-1502	Li N2 W	W	146
00-050-1482	Li0.84 N2 W1.16	W	194
01-089-7046	Li0.84 N2 W1.16	W	194
00-057-0399	Li6 N4 W	W	137
01-079-1123	Li6 N4 W	W	137

01-074-9122	Li6 N4 W	W	137
01-072-1288	Li N Zn	Zn	216
00-006-0467	Li Zn N	Zn	216
01-080-8523	Li N2 Zr	Zr	164
00-025-0506	Li2 N2 Zr	Zr	164
00-050-0843	Li2 N2 Zr	Zr	164
01-072-0793	Li2 N2 Zr	Zr	164
01-089-0306	Li2 N2 Zr	Zr	164

The number of unique ternaries were estimated by counting the number of unique spacegroups reported for each metal M (Table S7). We did not count PDF entries for which a spacegroup was not reported. If there are multiple competing reports for the same phase (e.g., experimental ambiguity in structural determination), then our counts may be overestimates. Additionally, the count of 10 unique structures for M = Na struck us as an outlier. These 10 unique structures originated from a single computational study.[8] As these phases have not been experimentally determined, we marked the count for Li-Na-N ternaries as 0 in Figure 6. Other computationally-derived structures may be present in Table S6 that we have not identified.

Table S7: Summary of unique space groups for Li-M-N ternaries shown in Table S6. Excluding Na, there are 63 unique Li-M-N phases reported.

Metal	Unique space groups
Al	1
B	4
Ba	2
Be	1
Ca	2
Ce	1
Co	1
Cr	2
Cu	2
Fe	3
Ga	1
Ge	2
Hf	1
Mg	3
Mn	3
Mo	3
Na	10
Nb	2
Ni	4
Re	1
Sc	1
Si	5
Sr	2
Ta	4
Th	1
Ti	1
U	2
V	3
W	3
Zn	1
Zr	1

Synthesis set-up

As described in the methods section, Li_2MN_2 (M = Zr, Hf) was synthesized under flowing nitrogen in a tube furnace. Air-free transfer of the process tube between the furnace and the glovebox was made possible via custom end caps with quick-connect attachments for the gas and exhaust lines (Figure S18a). Syntheses of $MgMN_2$ were conducted in sealed ampules in a muffle furnace (Figure S18b). These ampules were flame-sealed without exposure to air by using vacuum fittings for transfer between the glovebox and the sealing line.

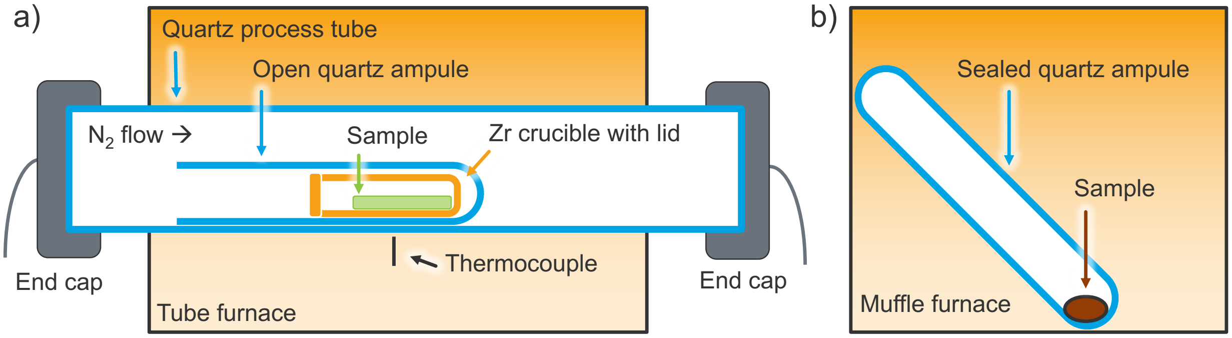

Figure S18: Diagrams showing a) the tube furnace configuration for the synthesis of Li_2MN_2 (M = Zr, Hf) and b) the muffle furnace configuration for the synthesis of $MgMN_2$.

References

(1) Williams, D. B. G.; Lawton, M. Drying of organic solvents: quantitative evaluation of the efficiency of several desiccants. *The Journal of organic chemistry* **2010**, *75*, 8351–8354.

(2) Todd, P. K.; Fallon, M. J.; Neilson, J. R.; Zakutayev, A. Two-step solid-state synthesis of ternary nitride materials. *ACS Materials Letters* **2021**, *3*, 1677–1683.

(3) Rom, C. L. et al. Mechanistically Guided Materials Chemistry: Synthesis of Ternary Nitrides, $CaZrN_2$ and $CaHfN_2$. *Journal of the American Chemical Society* **2024**, *146*, 4001–4012.

(4) Curtarolo, S.; Setyawan, W.; Hart, G. L.; Jahnatek, M.; Chepulskii, R. V.; Taylor, R. H.; Wang, S.; Xue, J.; Yang, K.; Levy, O.; Mehl, M. J.; Stokes, H. T.; Demchenko, D. O.; Morgan, D. AFLOW: An automatic framework for high-throughput materials discovery. *Computational Materials Science* **2012**, *58*, 218–226.

(5) Lederer, Y.; Toher, C.; Vecchio, K. S.; Curtarolo, S. The search for high entropy alloys: a high-throughput ab-initio approach. *Acta Materialia* **2018**, *159*, 364–383.

(6) Momma, K.; Izumi, F. VESTA 3 for three-dimensional visualization of crystal, volumetric and morphology data. *Journal of applied crystallography* **2011**, *44*, 1272–1276.

(7) Rumble, J. R. (ed.), "Standard Thermodynamic Properties of Chemical Substances" in CRC Handbook of Chemistry and Physics, 104th Edition (Internet Version). `https://hbcp.chemnetbase.com/documents/09_04/09_04_0001.xhtml?dswid=6263` (accessed 2024-08-26).

(8) Schön, J.; Wevers, M.; Jansen, M. Investigation of the possible ternary nitrides in the system Li_3N/Na_3N. *Solid state sciences* **2000**, *2*, 449–456.
